# Supplementary material for: Hepatotoxicity during 6-thioguanine treatment in inflammatory bowel disease and childhood acute lymphoblastic leukaemia: A systematic review
Source: PLoS One. 2019 May 24;14(5):e0212157. doi: 10.1371/journal.pone.0212157 (PMC6534292; doi:10.1371/journal.pone.0212157)
Supplement: S1 Appendix — (PDF) [file pone.0212157.s001.pdf]

Systematic review

1. * Review title.

Give the working title of the review, for example the one used for obtaining funding. Ideally the title should state succinctly the interventions or exposures being reviewed and the associated health or social problems. Where appropriate, the title should use the PI(E)COS structure to contain information on the Participants, Intervention (or Exposure) and Comparison groups, the Outcomes to be measured and Study designs to be included.

Hepatotoxicity during 6-thioguanine treatment: a systematic review

2. Original language title.

For reviews in languages other than English, this field should be used to enter the title in the language of the review. This will be displayed together with the English language title.

3. * Anticipated or actual start date.

Give the date when the systematic review commenced, or is expected to commence.

01/02/2018

4. * Anticipated completion date.

Give the date by which the review is expected to be completed.

01/01/2019

5. * Stage of review at time of this submission.

Indicate the stage of progress of the review by ticking the relevant Started and Completed boxes. Additional information may be added in the free text box provided.
Please note: Reviews that have progressed beyond the point of completing data extraction at the time of initial registration are not eligible for inclusion in PROSPERO. Should evidence of incorrect status and/or completion date being supplied at the time of submission come to light, the content of the PROSPERO record will be removed leaving only the title and named contact details and a statement that inaccuracies in the stage of the review date had been identified.
This field should be updated when any amendments are made to a published record and on completion and publication of the review. If this field was pre-populated from the initial screening questions then you are not able to edit it until the record is published.

The review has not yet started: No

Review stage	Started	Completed
Preliminary searches	Yes	Yes
Piloting of the study selection process	Yes	Yes
Formal screening of search results against eligibility criteria	Yes	Yes
Data extraction	Yes	No
Risk of bias (quality) assessment	No	No
Data analysis	No	No

Provide any other relevant information about the stage of the review here (e.g. Funded proposal, protocol not yet finalised).

Full-text screening completed, data extraction starting now.

Full-text screening completed, data extraction starting now.

6. * Named contact.

The named contact acts as the guarantor for the accuracy of the information presented in the register record.

Kjeld Schmiegelow

Email salutation (e.g. "Dr Smith" or "Joanne") for correspondence:

Professor Schmiegelow

7. * Named contact email.

Give the electronic mail address of the named contact.

kjeld.schmiegelow@regionh.dk

8. Named contact address

Give the full postal address for the named contact.

Department of Paediatrics and Adolescent Medicine

Rigshospitalet, University of Copenhagen

Blegdamsvej 9

2100 Copenhagen

Denmark

9. Named contact phone number.

Give the telephone number for the named contact, including international dialling code.

+45 3545 1357

10. * Organisational affiliation of the review.

Full title of the organisational affiliations for this review and website address if available. This field may be

completed as 'None' if the review is not affiliated to any organisation.

Department of Paediatrics and Adolescent Medicine, Rigshospitalet, University of Copenhagen,

Copenhagen, Denmark

Organisation web address:

https://www.rigshospitalet.dk/

11. * Review team members and their organisational affiliations.

Give the title, first name, last name and the organisational affiliations of each member of the review team. Affiliation refers to groups or organisations to which review team members belong.

Dr Linea Natalie Toksvang. Department of Paediatrics and Adolescent Medicine, Rigshospitalet, University of Copenhagen, Copenhagen, Denmark
Dr Rikke Hebo Larsen. Department of Paediatrics and Adolescent Medicine, Rigshospitalet, University of Copenhagen, Copenhagen, Denmark
Dr Thomas Leth Frandsen. Department of Paediatrics and Adolescent Medicine, Rigshospitalet, University of Copenhagen, Copenhagen, Denmark
Professor Kjeld Schmiegelow. Department of Paediatrics and Adolescent Medicine, Rigshospitalet, University of Copenhagen, Copenhagen, Denmark; Institute of Clinical Medicine, The Faculty of Medicine, University of Copenhagen, Denmark
Dr Cecilie Utke Rank. Department of Hematology, Rigshospitalet, University of Copenhagen, Copenhagen, Denmark; Pediatric Oncology Research Laboratory, Rigshospitalet, University of Copenhagen, Copenhagen, Denmark

12. * Funding sources/sponsors.

Give details of the individuals, organizations, groups or other legal entities who take responsibility for initiating, managing, sponsoring and/or financing the review. Include any unique identification numbers assigned to the review by the individuals or bodies listed.

The Danish Childhood Cancer Foundation and the Danish Cancer Society is supporting this work financially,

but the funders are not involved in the development of the protocol, the conduct of the review, the analysis

and interpretation of the data, or in the dissemination of the review

13. * Conflicts of interest.

List any conditions that could lead to actual or perceived undue influence on judgements concerning the main topic investigated in the review.

None

14. Collaborators.

Give the name and affiliation of any individuals or organisations who are working on the review but who are not listed as review team members.

15. * Review question.

State the question(s) to be addressed by the review, clearly and precisely. Review questions may be specific or broad. It may be appropriate to break very broad questions down into a series of related more specific questions. Questions may be framed or refined using PI(E)COS where relevant.

Primary objective: to assess the incidence of hepatotoxicity in patients treated with 6TG (6-thioguanine)

compared to 6MP (mercaptopurine) or standard care.

Secondary objective: to explore whether a safe dose of 6TG can be established.

16. * Searches.

Give details of the sources to be searched, search dates (from and to), and any restrictions (e.g. language or publication period). The full search strategy is not required, but may be supplied as a link or attachment.

Electronic searches:

We will systematically search the following databases:

- PubMed/MEDLINE (from 1998 to 2018 - the time limit was set at 1998 as 6TG was studied as a childhood ALL treatment in 1998, when two pilot trials compared 6TG to 6MP, and also to include the full period during which 6TG has been used in IBD treatment (since 20/01/14).

- Embase/Elsevier (from 1998 to 2018).

- Scopus/Elsevier (from 1998 to 2018).

- Web of Science Core Collection/Clarivate Analytics (from 1998 to 2018).

- The Cochrane Central Register of Controlled Trials (CENTRAL)/Wiley (The Cochrane Library, issue 2, 2018) (from 1998 to 2018).

Protocol amendment 20.03.18: Embase/Ovid was searched, since the Elsevier interface was not accesible at our centre.

The search strategies used for the abovementioned electronic databases will be made available in appendices to the review.

Other sources:

- The ‘Similar records’ option in Embase/Ovid, the ‘Related Articles’ option in PubMed/MEDLINE, and the ‘citation tracking’ feature in Web of Science Core Collection/Clarivate Analytics will be used.

In addition, we will also hand search the following:

- The reference lists of the included studies.

- Conference proceedings from:

- the American Society of Hematology (ASH) (from 1998 to 2018);

- European Hematology Association (EHA) (from 1998 to 2018);

- American College of Gastroenterology (ACG) (from 1998 to 2018);

- European Crohn’s and Colitis Organization (ECCO) (from 1998 to 2018);

- International Union of Basic & Clinical Pharmacology (IUPHAR) (from 1998 to 2018);

- European Association for Clinical Pharmacology and Therapeutics (EACPT) (from 1998 to 2018);

- ClinicalTrials.gov registry (www.ClinicalTrials.gov), the International Standard Randomized Controlled Trial Number (ISRCTN) registry (www.controlled-trials.com), WHO International Clinical Trials Registry Platform (ICTRP) (www.who.int/ictrp/search/en).

- PROSPERO will also be searched for ongoing systematic reviews.

Protocol amendment 8, 22.10.18: We planned on using the 'related articles' option in PubMed/MEDLINE, the 'similar records' option in Embase/Ovid, and the 'citation tracking' feature in Web of Science Core Collection/Clarivate Analytics, however we could not identify a feasible way to incorporate these measures.

The search will be developed and carried out by LNT and CUR.

17. URL to search strategy.

Give a link to a published pdf/word document detailing either the search strategy or an example of a search strategy for a specific database if available (including the keywords that will be used in the search strategies), or upload your search strategy.Do NOT provide links to your search results.

Alternatively, upload your search strategy to CRD in pdf format. Please note that by doing so you are consenting to the file being made publicly accessible.

Do not make this file publicly available until the review is complete

18. * Condition or domain being studied.

Give a short description of the disease, condition or healthcare domain being studied. This could include health and wellbeing outcomes.

Hepatotoxicity was recognised as a complication of 6-thioguanine (6TG) in 1976 (Griner et al, 1976). Since then, 6-TG associated hepatotoxicity in the form of acute sinusoidal obstruction syndrome (SOS), also known as veno-occlusive disease (VOD), has notably been reported in childhood acute lymphoblastic leukaemia (ALL) (Vora et al, 2006; Stork et al, 2010; Toksvang et al, 2017), whereas chronic nodular regenerative hyperplasia (NRH) has been reported in adults with inflammatory bowel disease (IBD) (Musumba, 2013) as well as in childhood ALL (Roy Moulik & Taj, 2017). However, SOS and NRH should be considered part of a spectrum of microvascular disorders caused by endothelial injury (DeLeve et al, 2002; DeLeve, 2011; Ghabril & Vuppalanchi, 2014). Nevertheless, the cellular mechanisms responsible for the sinusoidal damage, being pivotal to their development, remain to be established. 6TG-related SOS and NRH have been hypothesised to be dose-related, since they do not seem to occur with low cumulative doses (Oancea et al, 2013; de Boer et al, 2005).

References:

Griner, P.F., Elbadawi, A. & Packman, C.H. (1976) Veno-occlusive disease of the liver after chemotherapy of acute leukemia. Report of two cases. Annals of internal medicine, 85, 578–82.

Vora, A., Mitchell, C.D., Lennard, L., Eden, T.O.B., Kinsey, S.E., Lilleyman, J. & Richards, S.M. (2006) Toxicity and efficacy of 6-thioguanine versus 6-mercaptopurine in childhood lymphoblastic leukaemia: a randomised trial. Lancet, 368, 1339–48.

Stork, L.C., Matloub, Y., Broxson, E., La, M., Yanofsky, R., Sather, H., Hutchinson, R., Heerema, N. a, Sorrell, A.D., Masterson, M., Bleyer, A. & Gaynon, P.S. (2010) Oral 6-mercaptopurine versus oral 6-thioguanine and veno-occlusive disease in children with standard-risk acute lymphoblastic leukemia: report of the Children's Oncology Group CCG-1952 clinical trial. Blood, 115, 2740–8.

Toksvang, L.N., De Pietri, S., Nielsen, S.N., Nersting, J., Albertsen, B.K., Wehner, P.S., Rosthøj, S., Lähteenmäki, P.M., Nilsson, D., Nystad, T.A., Grell, K., Frandsen, T.L. & Schmiegelow, K. (2017) Hepatic sinusoidal obstruction syndrome during maintenance therapy of childhood acute lymphoblastic leukemia is associated with continuous asparaginase therapy and mercaptopurine metabolites. Pediatric Blood & Cancer, 64, e26519.

Musumba, C.O. (2013) Review article: the association between nodular regenerative hyperplasia, inflammatory bowel disease and thiopurine therapy. Alimentary pharmacology & therapeutics, 38, 1025–37.

Roy Moulik, N. & M Taj, M. (2017) Long-term risk of portal hypertension and related complications in children treated with 6-thioguanine for acute lymphoblastic leukemia: A single-center experience. Pediatric Blood & Cancer, 64, e26495.

DeLeve, L.D., Shulman, H.M. & McDonald, G.B. (2002) Toxic injury to hepatic sinusoids: sinusoidal obstruction syndrome (veno-occlusive disease). Seminars in Liver Disease, 22, 27–42.

DeLeve, L.D. (2011) Vascular Liver Disease. In Vascular liver disease: Mechanisms and management, DeLeve LD & Garcia-Tsao G (eds) pp 25–40. New York, NY: Springer New York.

Ghabril, M. & Vuppalanchi, R. (2014) Drug-induced nodular regenerative hyperplasia. Seminars in liver disease, 34, 240–5.

Oancea, I., Png, C.W., Das, I., Lourie, R., Winkler, I.G., Eri, R., Subramaniam, N., Jinnah, H.A., McWhinney, B.C., Levesque, J.-P., McGuckin, M.A., Duley, J.A. & Florin, T.H.J. (2013) A novel mouse model of veno-occlusive disease provides strategies to prevent thioguanine-induced hepatic toxicity. Gut, 62, 594–605.

de Boer, N.K.H., Mulder, C.J.J. & van Bodegraven, A.A. (2005) Nodular regenerative hyperplasia and thiopurines: the case for level-dependent toxicity. Liver transplantation : official publication of the American Association for the Study of Liver Diseases and the International Liver Transplantation Society, 11, 1300–1.

19. * Participants/population.

Give summary criteria for the participants or populations being studied by the review. The preferred format includes details of both inclusion and exclusion criteria.

Patients of any age and with any disease treated with 6TG.

Protocol amendment 3d 27.04.19: After completing the screening process we concluded that the majority of studies using 6TG for an extended period of time either as monotherapy or as part of maintenance treatment

was within ALL and IBD, we chose to limit the review to these two disease populations in order to make the review more focused.

20. * Intervention(s), exposure(s).

Give full and clear descriptions or definitions of the nature of the interventions or the exposures to be reviewed.

Treatment with 6TG.

Protocol amendment 20.03.18: During our literature screening we have encountered numerous protocols using 6TG as part of intensive chemotherapy regimens together with multiple other chemotherapeutics. Since it will not be feasible to evaluate the influence of 6TG in this setting, we have introduced a new exclusion criteria: "6-thioguanine only given as patr of an intensive chemotherapy phase concomitant with multiple other chemotherapeutics (ie. induction, consolidation, intensification, reinduction, block therapy, or conditioning regimens for bonw marrow transplantation)." We will include the study if 6TG was given as part of maintenance treatment.

21. * Comparator(s)/control.

Where relevant, give details of the alternatives against which the main subject/topic of the review will be compared (e.g. another intervention or a non-exposed control group). The preferred format includes details of both inclusion and exclusion criteria.

6MP or standard care (other non-6TG treatment regimen).

22. * Types of study to be included.

Give details of the types of study (study designs) eligible for inclusion in the review. If there are no restrictions on the types of study design eligible for inclusion, or certain study types are excluded, this should be stated. The preferred format includes details of both inclusion and exclusion criteria.

- Randomised controlled trials (RCTs) including quasi-randomised trials.
- Controlled clinical trials (CCTs).

- Observational studies (e.g. prospective and retrospective cohort studies, case-control, and cross-sectional studies).

- Case series and case reports, these will be reported separately.

23. Context.

Give summary details of the setting and other relevant characteristics which help define the inclusion or exclusion criteria.

There will be no restrictions by types of setting.

24. * Main outcome(s).

Give the pre-specified main (most important) outcomes of the review, including details of how the outcome is defined and measured and when these measurement are made, if these are part of the review inclusion criteria.

- The incidence of any hepatotoxicity reported by the studies as SOS, VOD, NRH, discordant thrombocytopenia, drug-induced liver injury or unspecified hepatotoxicity.

If an incidence rate, is not available, we will calculate it using prevalence and total follow-up time as reported

in the individual studies.

Because of the lack of standardised definitions of hepatotoxicity, authors of the included studies may not have used the above mentioned terms.

To assess further hepatotoxicity we will:

- Report any pathological findings of liver biopsies.
- Use the Ponte di Legno (PdL) toxicity working group consensus criteria for SOS, which entail fulfilment of at least three out of the following five criteria: (i) hyperbilirubinaemia; (ii) hepatomegaly; (iii) ascites; (iv) weight gain 5% or more; (v) thrombocytopenia (transfusion-resistant and/or unexplained by treatment) (Schmiegelow et al, 2016).
- Define hepatotoxicity as an increase of over two times upper normal limit in alanine transaminase or conjugated bilirubin, or a combined increase in aspartate transaminase, alkaline phosphatase, and total bilirubin provided one of them is above two times upper normal limit (Gisbert et al, 2007).

References:

Schmiegelow, K., Attarbaschi, A., Barzilai, S., Escherich, G., Frandsen, T.L., Halsey, C., Hough, R., Jeha, S., Kato, M., Liang, D.-C., Mikkelsen, T.S., Möricke, A., Niinimäki, R., Piette, C., Putti, M.C., Raetz, E., Silverman, L.B., Skinner, R., Tuckuviene, R., van der Sluis, I., et al (2016) Consensus definitions of 14 severe acute toxic effects for childhood lymphoblastic leukaemia treatment: a Delphi consensus. The Lancet Oncology, 17, e231-9.

Gisbert, J.P., González-Lama, Y. & Maté, J. (2007) Thiopurine-induced liver injury in patients with inflammatory bowel disease: a systematic review. The American journal of gastroenterology, 102, 1518–27.

Timing and effect measures

Since the duration of 6TG treatment in relation to development of hepatotoxicity as well as the time to hepatotoxicity after 6TG exposure are unknown, no restrictions regarding timing will be applied.

25. * Additional outcome(s).

List the pre-specified additional outcomes of the review, with a similar level of detail to that required for main outcomes. Where there are no additional outcomes please state ‘None’ or ‘Not applicable’ as appropriate to the review

- Diagnostic methods: the numbers of patients who had a liver biopsy, indications for a liver biopsy, received other diagnostic procedures, and the study conclusions regarding these diagnostic methods
- Dose reduction or truncation of 6TG due to hepatotoxicity, as registered in the data: how many patients had their 6TG truncated, or their doses reduced (doses before and after dose reduction will be extracted).

Timing and effect measures

26. * Data extraction (selection and coding).

Give the procedure for selecting studies for the review and extracting data, including the number of

researchers involved and how discrepancies will be resolved. List the data to be extracted.

Literature search results will be uploaded to Covidence systematic review software, Veritas Health Innovation, Melbourne, Australia, for study selection, and will additionally be used to remove any duplicates. We will aim to avoid double counting by juxtaposing author names, locations and settings, and sample sizes. Study authors will be contacted by email to resolve any uncertainties.

Selection of studies:

Two review authors (LNT and CUR) will independently screen the titles and abstracts yielded by the searches against the inclusion criteria. Eligible studies will be evaluated as full texts by both authors, and decisions made regarding final inclusion. Any disagreements will be resolved through discussion between LNT and CUR, and unresolved disagreements will be adjudicated by KS. The reasons for excluding any studies will be recorded, and the review authors will not be blinded to the journal titles, study authors or institutions.

Data extraction and management:

Two review authors (LNT and CUR) will independently extract data from included studies in duplicate using a standardised extraction form, which will be approved by all authors. Disagreements will be resolved through discussion between LNT and CUR, and unresolved disagreements will be adjudicated by KS.

Protocol amendment 4, 27.04.18: We included two new authors, Sofie Arup and Magnus Støh Schmidt, in the study group. Two review authors (SA and MSS) will independently extract data from included studies in duplicate using a standardised extraction form, which will be approved by all authors. Disagreement will be resolved through discussion between SA and MSS; unresolved disagreement will be adjudicated by LNT.

Data items:

We will extract information on the authors and the year of publication, the study characteristics (design, blinding, setting, duration, inclusion and exclusion criteria, sources of funding, conflicts of interest, ethical approvals, and the key conclusions of the study authors), the patient characteristics (the number of patients, their ages, genders, ethnicities, diseases, comorbidities, and any concomitant therapies), details of the interventions (the duration of the 6TG, the dose of 6TG, the cumulative dose of 6TG, the maximum dose of 6TG, the route of administration, and the ery-TGN levels), the comparators (comparator drugs, the duration of 6MP or other standard care drug, the dose of 6MP or other standard care drug, the cumulative dose of 6MP or other standard care drug, the maximum dose of 6MP or other drug, and the route of administration), follow-up, and risk of bias.

27. * Risk of bias (quality) assessment.

State whether and how risk of bias will be assessed (including the number of researchers involved and how discrepancies will be resolved), how the quality of individual studies will be assessed, and whether and how this will influence the planned synthesis.

The Cochrane Collaboration tool for assessing the risk of bias (Higgins & Green) will be applied to the RCTs included in this review. Two review authors (LNT and CUR) will independently collect information on: (1) sequence generation, (2) allocation concealment, (3) blinding of participants and personnel, (4) blinding of outcome assessors, (5) incomplete outcome data, (6) selective outcome reporting, and (7) other bias including baseline imbalance, early stopping, bias due to financial interest, and academic bias. A judgement of the possible risk of bias on each of the seven domains will be given as 'low risk' or 'high risk'. In the case of insufficient reporting, the risk of bias will be judged as "unclear", and study authors will be contacted for additional information. Reviewers will not be blinded to the studies, and disagreements will be resolved through discussion between LNT and CUR, with any unresolved disagreements being adjudicated by KS. A graphic illustration of potential bias will be presented using RevMan 5.3 (Review Manager 5.3) software, if possible.

Non-randomised studies included in the review will be evaluated for potential bias using the ROBINS-I tool provided by the Cochrane Collaboration (Sterne et al, 2016). Pre-specified confounders include: age, gender, comorbidity (including liver disease) and the concomitant use of hepatotoxic drugs.

Two review authors (LNT and CUR) will independently collect information on bias due to: (1) confounding, (2) selection of participants, (3) classification of events, (4) classification of interventions, (5) deviations from intended interventions, (6) missing data, (7) measurement of outcomes, and (8) selection of the reported result. A judgement of the possible risk of bias on each of the eight domains will be rated as 'low risk', 'moderate risk', 'serious risk', 'critical risk' or 'no information'. In the case of insufficient reporting, study authors will be contacted and additional information requested. Disagreements will be resolved through discussion between LNT and CUR, and unresolved disagreements will be adjudicated by KS. Reviewers will, in addition, not be blinded to studies.

Studies with a low risk of bias will be ascribed more weight in the overall findings of the review compared to studies with a higher risk of bias.

Protocol amendment 5, 27.04.18: All assessments of bias will be carried out independently by SA and MSS. Disagreement will be resolved through discussion between SA and MSS; unresolved disagreement will be adjudicated by LNT.

Protocol amendment 7, 12.10.18: Instead of ROBINS-I, we decided to use the Cochrane Quality Assessment Tool for Observational Cohort and Cross-Sectional Studies to evaluate the bias of observational studies because it was better suited for the included observational studies which generally did not have both an intervention and comparator group, but were mostly cohort studies.

Protocol amendment 9, 11.12.18: Correction to amenment 7: The study quality assessment tools of the National Heart, Lung, and Blood Institute of the National Institutes of Health (NIH) for quality assessment of Observational Cohort and Cross-Sectional Studies and Controlled Intervention Studies were used to assess the risk of bias of non-randomised studies.

Meta-biases:

We will address publication bias by searching for and including grey literature. To address selective outcome bias, we will compare outcomes between protocols and published reports, and if protocols are not available, we will compare the outcomes reported in the methods and results sections. No analyses will be carried out to assess the impact of meta-biases, as we will not be providing a quantitative synthesis.

Confidence in cumulative evidence:

The Grading of Recommendations, Assessment, Development and Evaluation (GRADE) approach (Atkins et al, 2004) will be used to assess the confidence in the cumulative evidence. Two review authors (LNT and CUR) will independently evaluate the quality of evidence in each of four domains: (1) study design, (2) study quality, (3) consistency, and (4) directness. The evidence on each outcome will be graded as ‘very low’, ‘low’, ‘moderate’ or ‘high’. Disagreements will be resolved through discussion between LNT and CUR, with any unresolved disagreements being adjudicated by KS.

Protocol amendment 6, 27.04.18: The GRADE assessment will be carried out independently by SA and MSS. Disagreement will be resolved through discussion between SA and MSS; unresolved disagreement will be adjudicated by LNT.

28. * Strategy for data synthesis.

Give the planned general approach to synthesis, e.g. whether aggregate or individual participant data will be used and whether a quantitative or narrative (descriptive) synthesis is planned. It is acceptable to state that a quantitative synthesis will be used if the included studies are sufficiently homogenous.

Assessment of heterogeneity:

A forest plot will be used to summarise the findings of the included studies and to assess heterogeneity between studies, the plot being sorted by study design. Meta-regression analyses will not be performed.

Data synthesis:

Based on the preliminary searches, we do not expect the studies on this subject to be sufficiently homogenous in terms of design and comparators to enable a quantitative data synthesis to be carried out, and hence, a systematic narrative synthesis of the characteristics and findings of the included studies will be provided instead with the information presented in text, tables, and as a graphical presentation of SOS/NRH

incidence compared to 6TG dose in the included studies.

29. * Analysis of subgroups or subsets.

Give details of any plans for the separate presentation, exploration or analysis of different types of participants (e.g. by age, disease status, ethnicity, socioeconomic status, presence or absence or co-morbidities); different types of intervention (e.g. drug dose, presence or absence of particular components of intervention); different settings (e.g. country, acute or primary care sector, professional or family care); or different types of study (e.g. randomised or non-randomised).

Patients with known liver disease will be subject to sub-analyses, as liver disease may entail an inherently higher risk of hepatotoxicity (DeLeve, 2011).

References:

DeLeve, L.D. (2011)Vascular Liver Disease. In Vascular liver disease: Mechanisms and management, DeLeve LD & Garcia-Tsao G (eds) pp 25–40. New York, NY: Springer New York.

30. * Type and method of review.

Select the type of review and the review method from the lists below. Select the health area(s) of interest for your review.

Type of review

Cost effectiveness
No

Diagnostic
No

Epidemiologic
No

Individual patient data (IPD) meta-analysis
No

Intervention
Yes

Meta-analysis
No

Methodology
No

Narrative synthesis
No

Network meta-analysis
No

Pre-clinical
No

Prevention
No

Prognostic
No

Prospective meta-analysis (PMA)
No

Review of reviews
No

Service delivery

No

Synthesis of qualitative studies
No

Systematic review
Yes

Other
No

Health area of the review

Alcohol/substance misuse/abuse
No

Blood and immune system
Yes

Cancer
Yes

Cardiovascular
Yes

Care of the elderly
No

Child health
Yes

Complementary therapies
No

Crime and justice
No

Dental
No

Digestive system
Yes

Ear, nose and throat
No

Education
No

Endocrine and metabolic disorders
No

Eye disorders
No

General interest
No

Genetics
No

Health inequalities/health equity
No

Infections and infestations
No

International development
No

Mental health and behavioural conditions
No

Musculoskeletal

No

Neurological
No

Nursing
No

Obstetrics and gynaecology
No

Oral health
No

Palliative care
No

Perioperative care
No

Physiotherapy
No

Pregnancy and childbirth
No

Public health (including social determinants of health)
No

Rehabilitation
No

Respiratory disorders
No

Service delivery
No

Skin disorders
No

Social care
No

Surgery
No

Tropical Medicine
No

Urological
No

Wounds, injuries and accidents
No

Violence and abuse
No

31. Language.

Select each language individually to add it to the list below, use the bin icon to remove any added in error.
English

There is an English language summary.

32. Country.

Select the country in which the review is being carried out from the drop down list. For multi-national collaborations select all the countries involved.

Denmark

33. Other registration details.

Give the name of any organisation where the systematic review title or protocol is registered (such as with The Campbell Collaboration, or The Joanna Briggs Institute) together with any unique identification number assigned. (N.B. Registration details for Cochrane protocols will be automatically entered). If extracted data will be stored and made available through a repository such as the Systematic Review Data Repository (SRDR), details and a link should be included here. If none, leave blank.

34. Reference and/or URL for published protocol.

Give the citation and link for the published protocol, if there is one

Give the link to the published protocol.

Alternatively, upload your published protocol to CRD in pdf format. Please note that by doing so you are consenting to the file being made publicly accessible.

No I do not make this file publicly available until the review is complete

Please note that the information required in the PROSPERO registration form must be completed in full even if access to a protocol is given.

35. Dissemination plans.

Give brief details of plans for communicating essential messages from the review to the appropriate audiences.

We plan to publish this systematic review in an international peer-reviewed journal.

Do you intend to publish the review on completion?

Yes

36. Keywords.

Give words or phrases that best describe the review. Separate keywords with a semicolon or new line. Keywords will help users find the review in the Register (the words do not appear in the public record but are included in searches). Be as specific and precise as possible. Avoid acronyms and abbreviations unless these are in wide use.

Thioguanine; Hepatotoxicity; Sinusoidal-obstruction syndrome; SOS; Nodular regenerative hyperplasia;

NRH; Drug-induced liver injury; DILI

37. Details of any existing review of the same topic by the same authors.

Give details of earlier versions of the systematic review if an update of an existing review is being registered, including full bibliographic reference if possible.

38. * Current review status.

Review status should be updated when the review is completed and when it is published. For newregistrations the review must be Ongoing.
Please provide anticipated publication date

Review_Ongoing

39. Any additional information.

Provide any other information the review team feel is relevant to the registration of the review.

The present protocol has been prepared in accordance with the Cochrane Handbook (Higgins & Green) and the Preferred Reporting Items for Systematic Reviews and Meta-Analyses Protocols (PRISMA-P) statement (Shamseer et al, 2015)

The systematic review will be conducted in accordance with the Cochrane Handbook (Higgins & Green) and PRISMA statement (Moher et al, 2009).

Amendments

In the event of protocol amendments, each amendment will be listed with date, description of the change and rationale behind it.

References:

Higgins, J. & Green, S. Cochrane Handbook for Systematic Reviews of Interventions Version 5.1.0 [updated March 2011]. The Cochrane Collaboration, 2011. Available at: www.cochrane-handbook.org.

Shamseer, L., Moher, D., Clarke, M., Ghersi, D., Liberati, A., Petticrew, M., Shekelle, P., Stewart, L.A. & PRISMA-P Group (2015) Preferred reporting items for systematic review and meta-analysis protocols (PRISMA-P) 2015: elaboration and explanation. BMJ (Clinical research ed.), 350, g7647.

Moher, D., Liberati, A., Tetzlaff, J., Altman, D.G. & PRISMA Group (2009) Preferred Reporting Items for Systematic Reviews and Meta-Analyses: The PRISMA Statement. PLoS Medicine, 6, e1000097.

Sterne, J.A., Hernán, M.A., Reeves, B.C., Savovi?, J., Berkman, N.D., Viswanathan, M., Henry, D., Altman, D.G., Ansari, M.T., Boutron, I., Carpenter, J.R., Chan, A.-W., Churchill, R., Deeks, J.J., Hróbjartsson, A., Kirkham, J., Jüni, P., Loke, Y.K., Pigott, T.D., Ramsay, C.R., et al (2016) ROBINS-I: a tool for assessing risk of bias in non-randomised studies of interventions. BMJ (Clinical research ed.), 355, i4919.

Atkins, D., Best, D., Briss, P.A., Eccles, M., Falck-Ytter, Y., Flottorp, S., Guyatt, G.H., Harbour, R.T., Haugh, M.C., Henry, D., Hill, S., Jaeschke, R., Leng, G., Liberati, A., Magrini, N., Mason, J., Middleton, P., Mrukowicz, J., O’Connell, D., Oxman, A.D., et al (2004) Grading quality of evidence and strength of recommendations. BMJ (Clinical research ed.), 328, 1490.

40. Details of final report/publication(s).

This field should be left empty until details of the completed review are available.

Give the link to the published review.
