# Supplementary material for: Hepatotoxicity during 6-thioguanine treatment in inflammatory bowel disease and childhood acute lymphoblastic leukaemia: A systematic review
Source: PLoS One. 2019 May 24;14(5):e0212157. doi: 10.1371/journal.pone.0212157 (PMC6534292; doi:10.1371/journal.pone.0212157)
Supplement: S2 Appendix — (PDF) [file pone.0212157.s002.pdf]

S2 Appendix. Full search strategy

Search strategies:

Electronic searches	Search strategy	Number of hits
PubMed	Thioguanine [mh] OR thioguanine [ot] OR thioguanine [ti] OR tioguanine [ot] OR tioguanine [ti] OR 6-thioguanine [ot] OR 6-thioguanine [ti] OR 6-tioguanine [ti] OR 6-tioguanine [ot] OR 6-TG [ot] OR 6-TG [ti] OR 6TG[ot] OR 6TG [ti] AND 1998:2018[dp]	*792*
Embase	*thioguanine/ OR (thioguanine or tioguanine or 6-thioguanine or 6thioguanine or 6-tioguanine or 6-TG or 6TG).ti,kw. and 1998:20018.(sa.year)	*769*
Scopus	TITLE(thioguanine) OR AUTHKEY(thioguanine) OR TITLE(tioguanine) OR AUTHKEY (tioguanine) OR TITLE(6-thioguanine) OR AUTHKEY(6-thioguanine) OR TITLE(6thioguanine) OR AUTHKEY(6thioguanine) OR TITLE(6-tioguanine) OR AUTHKEY(6-tioguanine) OR TITLE(6-TG) OR AUTHKEY(6-TG) OR TITLE(6TG) OR AUTHKEY(6TG) AND PUBYEAR > 1997	*576*
Web of Science Core Collection	TS=(t$ioguanine) OR TS=(6-t$ioguanine) OR TS=(6t$ioguanine) OR TS=(6-TG) OR TS=(6TG) *Indexes=SCI-EXPANDED, SSCI, A&HCI, CPCI-S, CPCI-SSH, BKCI-S, BKCI-SSH, ESCI Timespan=1998-2018* Limitations*	*1087*
Conference proceedings		
American Society of Hematology (ASH)	thioguanine, tioguanine, 6-thioguanine, 6thioguanine, 6-tioguanine, 6-TG, 6TG	*15*
European Hematology Association (EHA)	thioguanine, tioguanine, 6-thioguanine, 6thioguanine, 6-tioguanine, 6TG, 6-TG	*28*
American College of Gastroenterology (ACG)	thioguanine, tioguanine, 6-thioguanine, 6thioguanine, 6-tioguanine, 6TG, 6-TG	*2*
European Crohn's and Colitis Organization (ECCO)	thioguanine, tioguanine, 6-thioguanine, 6thioguanine, 6-tioguanine, 6TG, 6-TG	*15*
American Association for the Study of Liver Diseases (AASLD)	thioguanine, tioguanine, 6-thioguanine, 6thioguanine, 6-tioguanine, 6TG, 6-TG	*0*
International Union of Basic & Clinical Pharmacology (IUPHAR)	thioguanine, tioguanine, 6-thioguanine, 6thioguanine, 6-tioguanine, 6TG, 6-TG	*5*
European Association for Clinical Pharmacology and Therapeutics (EACPT)	thioguanine, tioguanine, 6-thioguanine, 6thioguanine, 6-tioguanine, 6TG, 6-TG	*0*
International Prospective Register of	thioguanine OR tioguanine OR 6-thioguanine OR 6thioguanine OR 6-tioguanine OR 6-TG OR 6TG	*4*

Systematic Reviews (PROSPERO)		

* **Refined by: WEB OF SCIENCE CATEGORIES:** (GASTROENTEROLOGY HEPATOLOGY OR PHARMACOLOGY PHARMACY OR ONCOLOGY OR HEMATOLOGY OR OBSTETRICS GYNECOLOGY OR NEUROSCIENCES OR CLINICAL NEUROLOGY OR GERIATRICS GERONTOLOGY OR PEDIATRICS OR OPHTHALMOLOGY OR RHEUMATOLOGY OR DERMATOLOGY OR SURGERY OR UROLOGY NEPHROLOGY OR TRANSPLANTATION OR INFECTIOUS DISEASES OR PATHOLOGY OR RADIOLOGY NUCLEAR MEDICINE MEDICAL IMAGING)
