# Supplementary material for: Hepatotoxicity during 6-thioguanine treatment in inflammatory bowel disease and childhood acute lymphoblastic leukaemia: A systematic review
Source: PLoS One. 2019 May 24;14(5):e0212157. doi: 10.1371/journal.pone.0212157 (PMC6534292; doi:10.1371/journal.pone.0212157)
Supplement: S3 Appendix — (PDF) [file pone.0212157.s003.pdf]

S3 Appendix. Detailed description of included studies

Study characteristics of the included RCTs are presented in Table A, study characteristics of observational studies in Table B, and study characteristics of case report/case series in Table C. Results of the included RCTs are presented in Table D, results of observational studies in Table E, and results of case report/case series in Table F.

Randomised controlled trials

We included four RCTs. One RCT on relapsed childhood ALL used 6TG at a dose of 50 mg/m^2/day together with weekly methotrexate as maintenance therapy for 24 months in 157 children, and for 12 months in 26 children. No hepatotoxicity was reported in either treatment arm at 15-year follow-up. [1] There was no comparator to 6TG.

Three other RCTs on childhood ALL compared maintenance therapy with 6TG to 6MP (target dose 50–75 mg/m^2/day). In the CCG-1952 trial, the 6TG starting dose was reduced from 60 to 50 mg/m^2/day by February 1998, [2] while the COALL-92 trial reduced the 6TG starting dose from 50 to 40 mg/m^2/day – in both cases due to thrombocytopenia. [3] A starting dose of 40 mg/m^2/day was used throughout the UK MRC ALL97/99 trial. [4] SOS was reported in the UK MRC ALL97/99 and CCG-1952 trials in 9% (N=68) and 25% (N=257) of patients in the 6TG-arm, respectively. [2,4] Both trials used clinical criteria to diagnose SOS fulfilling two of the following; hyperbilirubinaemia, hepatomegaly, discordant thrombocytopenia and/or ascites. The COALL-92 trial did not specifically report SOS; however, a 7.5 fold higher incidence of discordant thrombocytopenia was reported in the 6TG-arm. [3] Liver biopsies were obtained from 12 patients from the CCG-1952 trial, among whom 10 had hepato-portal sclerosis, one had venous dilatation, and one was normal. [5] Liver biopsies from 10 patients in the UK MRC ALL97/99 trial all showed

mild portal fibrosis or NRH. [4] In the CCG-1952 trial, Doppler ultrasound (US) was performed in 168 patients; showing ascites in 70 patients and reversal of portal flow in 11 patients. Three patients developed hepatic failure, of whom one had a liver transplant. [2] MRI/MRA or upper gastrointestinal (GI) endoscopy showed oesophageal and/or gastric varices in 11 cases, and three patients had actively bleeding esophageal varices.[5] In the UK MRC ALL97/99 trial, Doppler US was conducted in 10 patients with discordant thrombocytopenia; showing SOS in four patients, possible SOS in three patients, and was normal in three patients. [6] Upper GI endoscopy in 12 patients with suspected portal hypertension from this trial showed oesophageal varices in seven cases. One patient required liver transplantation because of hepatopulmonary syndrome. [4]

Observational studies

Four of the 20 included observational studies investigated the pharmacokinetics of 6TG. Together these studies encompassed a total of 47 patients and did not report any hepatotoxicity. All these studies noted a substantial inter-individual variation in the pharmacokinetics of 6TG. [7–10]

Most observational studies focused on IBD, except those reporting subcohorts from the RCTs investigating childhood ALL. Of the ALL trials, the CCG-1942 trial (58 patients; pilot study to the CCG-1952 trial) used 6TG orally (60mg/m^2/day) as well as a continuous intravenous infusion (CIVI) (480 mg/m^2 over 24 hours every eight weeks), and reported SOS in 10% and alanine transaminase elevation during CIVI in 41%. At nine years follow-up, one patient (2%) showed evidence of portal hypertension. [11] A subcohort from the CCG-1952 trial reported SOS in 23% (N=351). [12] In the latest report from the MRC UKALL 97/99 trial (6TG dose 40mg/m^2) hepatotoxicity was reported in 19% (N=59) of 6TG-treated patients in one centre, with NRH in four patients, oesophageal varices in four patients, and a follow-up of 107 patient-years. [13] In one trial,

using 6TG at 50 mg/m^2 together with methotrexate and vincristine for three months for relapsed childhood ALL, no hepatotoxicity was reported during a four year-follow-up period. [14]

A conservative estimate of the number of patients included in observational studies concerning adult IBD patients treated with 6TG is at least 794 patients in total. Hepatotoxicity in IBD patients treated with 6TG was first reported in 2002 in a cohort of 111 patients who received 6TG doses of 40 (10–100) mg/day and cumulative doses of 1.2–36 g. Elevated liver function tests occurred in 24% (N=27), while NRH was documented by liver biopsy in 14% (N=16). [15] Ery-TGN levels did not differ between patients with and without NRH or laboratory abnormalities. [16,17]

One study measured the hepatic venous pressure gradient (HVPG) in 26 patients treated with 6TG at 10 (6–40) mg/day at the time of HVPG measurement. These patients had received a maximum dose of 40 (20–80) mg/day over a period of 38 (12–45) months prior to the HVPG measurement. Liver biopsy in 24 of the patients showed NRH in 25% (N=6). HVPG was 7 (3–14) mmHg in patients with NRH, and 3 (2–5) mmHg in patients without NRH. [18]

A systematic survey identified 296 patients treated with 6TG for 370 patient-years between 2001 and 2004 in eight countries. 6TG was commenced at 40 mg/day, but were 20 (20–40) mg/day in patients without discontinuation after 93 weeks. Elevated liver enzymes were found in 15% (N=43); liver biopsy was performed in 60 of the patients, and showed acute hepatotoxicity in 13% (N=8), chronic hepatotoxicity in 38% (N=23), NRH in 10% (N=6), and possible NRH in 17% (N=10). Computed tomography (CT) scans were conducted in 16 patients, MRI in 53 patients, and ultrasound in 149 patients, and showed splenomegaly in 39% (N=68) and NRH in 1% (N=2). The individual investigators decided whether to perform any imaging and/or liver biopsy. 6TG was truncated in seven patients, among whom three patients with elevated liver enzymes failed to recover by the end of the follow-up period of 39 (13–113) weeks. [19]

Less hepatotoxicity was observed in a retrospective cohort of 62 patients treated between 2009 and 2013, when the daily dose of 6TG was split in two or more doses, with daily doses of 0.6 (0.3–1.0) mg/kg but no individual dose exceeding 0.3 mg/kg. A liver biopsy was obtained in 11 asymptomatic patients, who were treated at the same centre, and showed NRH in one case. MRI was performed in two patients and US in 21 patients; all these examinations were normal. [20]

In a retrospective cohort of 111 patients treated between 2002 and 2014 in seven Dutch centres, 6TG was administered at a dose of 21 (standard deviation 5) mg/day - reaching a cumulative dose of 13 (4–39) g. A liver biopsy was obtained in all patients, and showed NRH in 6% (N=7), lesser degrees of nodularity in 11% (N=12), and sinusoidal dilatation in 85% (N=76). No clinical adverse events occurred during the 20 (7–64) months of follow-up. A CT scan was conducted in two patients and ultrasound in 76 of the patients, and showed splenomegaly in 4% (N=3). [21]

In another retrospective cohort of 54 patients treated for 126 patient-years between 2001 and 2013 with 6TG doses of 27 (20–40) mg/day and reaching a cumulative dose of 13 (interquartile range 4–26) g, portal hypertension was reported in one case, and elevated liver enzymes in two other cases. All reported hepatotoxicites resolved when 6TG was truncated. A liver biopsy was obtained in eleven patients as a screening after 12 months or more of 6TG therapy, and no evidence of NRH was noted in any case; MRI was similarly performed as a screening in 24 patients and showed splenomegaly in one case. [22]

Case reports

Thirteen of the 18 included case reports or case series encompassing 60 patients described hepatotoxicity defined as either abnormal liver enzymes, clinical SOS, or NRH. The reports that described hepatotoxicity used 6TG doses of 14–125 mg/m^2/day with cumulative doses up to 20 g, whereas those that did not report hepatotoxicity used 6TG doses of 0.8–21 mg/m^2/day and cumulative doses up to 19 g. Only five of the reports provided ery-TGN levels, which were 1156

pmol/8·10^8 RBC in one report that described hepatotoxicity, and 204–1143 pmol/8·10^8 RBC in the reports that did not note hepatotoxicity. In seven reports that described hepatotoxicity, a liver biopsy was performed in 16 patients, of which nine showed SOS, and four showed NRH. In one report that did not note hepatotoxicity, a liver biopsy was obtained in eight patients, in which no abnormalities were noted in five, subtle portal inflammation in one, slight sinusoidal dilatation in one, and steatosis in one case. Ultrasound was conducted in 16 patients across five reports that all noted hepatotoxicity, and found ascites in one, altered liver echogenicity in eight, and no abnormalities in four cases. Three reports that all reported hepatotoxicity used CT scans in three patients, of which two were normal and one showed hepatomegaly with mosaic pattern after dye injection. Two reports that both noted hepatotoxicity used magnetic resonance imaging (MRI) in 10 patients, which showed portal hypertension in one, gastric varices in one, increased contrast enhancement in one, and a diffuse patchy pattern in the portal phase (clover-like sign) in one. In six reports, 6TG treatment was truncated due to hepatotoxicity (SOS in three reports, NRH in one report, discordant thrombocytopenia in one report, and elevated liver enzymes in one report).

Two case reports describe 16 patients from the MRC UKALL 97/99 cohort in which clinically significant portal hypertension manifested by splenomegaly and thrombocytopenia persists in 13 cases. [23,24] Liver biopsy in six patients showed NRH in two cases. [23] Furthermore, endoscopy in 13 patients showed oesophageal varices in eight and gastric varices in one. [23,24] A case series from the CCG-1952 trial report hepatotoxicity in 12 cases, of which four developed chronic portal hypertension at 7–44 months after stopping 6TG. In all other cases the hepatotoxicity resolved completely. [25]

Table A. Study characteristics of included randomised controlled trials

Trial	Report	Year	Publication type	Setting	Duration	Total number of patients	Number of patients in 6TG group	Number of patients in control group	Age, years median (range)	Gender, % male	Concomitant therapy
ALL-REZ BFM 87	Einsiedel et al.[1]	2005	Original article	Multi-centre, Germany	4/1997–4/1990	207	Group A+B, 157	Group C, 26	<18	57	MTX
CCG-1952	Matloub et al.[26]	2006	Original article	Multi-centre, USA	5/1996 – 2/2000	2027	Not reported	Not reported	1–9	56	IT MTX or ITT, MTX, prednisone, VCR
	Stork et al.[27]	1998	Conference abstract			Not reported	Not reported	Not reported	Not reported	Not reported	
	Stork et al.[28]	2002	Conference abstract			2026	1016	1010	1–10	Not reported	
	Stork et al.[5]	2002	Conference abstract			2177	1012	1017	1–9.9	57	
	Stork et al.[2]	2010	Original article			2027	1017	1010	1–1.9, n=165; 2–5.9, n=1402; 6–9.9, n=460	56	
CO ALL-92	Erb et al.[29]*	1998	Original article	Single-centre, Germany	2/1992 – 8/1997	54	22	32	1–18	Not reported	MTX
	Harms et al.[30]	2000	Original article	Multi-centre, Germany		538	236	238	<1, n=17; 1–9, n=415; >10, n=104	58	
	Harms et al.[3]	2003	Original article			474	236	238	4.8 (1–17.8)	58	
UK MRC ALL 97/ Pilot	Lancaster et al.[31]	1998	Original article	Single-centre, UK	10/1995 – 6/1997	46	23	23	5 (1–16)	61	ASP, CYC, CYT, dexamethasone, MTX, VCR
	Stoneham et al.[6]	2003	Original article		10/1995 – 11/2001	99	62	37	1–14	57	Not reported
UK MRC ALL97 & UKALL XI	Hann et al.[32]	2000	Original article	Multi-centre, UK	3/1992 – 7/1998	1781	Not reported	Not reported	1–18	54	MTX, steroid (randomised: prednisone/dexamethasone), VCR
UK MRC ALL 97 & 97/99	Cartwright et al.[33]	2008	Conference abstract	Single-centre, UK	Not reported	80	34	46	1–18	55	MTX, steroid (randomised: prednisone/ dexamethasone), VCR
	Cartwright et al.[34]*	2011	Conference abstract		4/1997 – 4/2009	130	31	99	1–16	61	
	Lancaster et al.[35]*	2002	Original article		Not reported	10	6	4	6.2 (2.8–10.5)	50	
	Lennard et al.[36]	2001	Conference abstract		Not reported	12	8	4	7 (3–16)	70	
	Lennard et al.[37]	2003	Conference abstract	Multi-centre, UK and Ireland	1/1997 – 6/2002	1496	749	747	Not reported	Not reported	
	Lennard et al.[38]	2005	Conference abstract		1/1997 – 6/2003	1498	750	748	Not reported	Not reported	

	Lennard et al.[39]	2006	Original article		1/1997 – 6/2006	1492	748	744	4 (1–18)	Not reported	
	Lennard et al.[40]	2008	Conference abstract		1/1997 – 6/2004	1194	450	744	4 (1–18)	Not reported	
	Lennard et al.[41]	2008	Conference abstract		1/1997 – 6/2005	1194	450	744	Not reported	Not reported	
	Lennard et al.[42]*	2013	Original article		1/1997 – 6/2007	1211	457	754	1–18	Not reported	
	Lennard et al.[43]*	2015	Original article		Not reported – June 2002	1135	426	709	<2, n=74; 2-9, n=865; >10, n=143	53	
	Mitchell et al.[44]	2002	Conference abstract		1/1997 – 6/2007	1432	Not reported	Not reported	Not reported	Not reported	
	Mitchell et al.[45]	2009	Original article		1/1997 – 6/2007	1492	748	744	<2, n=139; 2-9, n=1396; >10n=400	56	
	Mitchell et al.[46]	2010	Original article		1980 – 2001	1440	723	717	<1, n=1; 1–9, n=1534; >10, n=400	56	
	Vora et al.[47]	2002	Conference abstract		1/1997 – 6/2002	1342	668	674	1–18	Not reported	
	Vora et al.[4]	2006	Original article		4/1997 – 6/2002	1492	748	744	<2, n=114; 2-9, n=1154; >10, n=224	54	

6TG, 6-thioguanine; ASP, asparaginase; CYC, cyclophosphamide; CYT, cytarabine; IT, intrathecal; ITT, intrathecal triple therapy; MTX, methotrexate; VCR, vincristine.

* Studies only concerning pharmacokinetics or TPMT activity

Table B. Study characteristics of included observational studies

Study	Report	Year	Publication type	Design	Setting	Duration	Total number of patients	Age (years), median (range)	Gender, % male	Disease	Concomitant therapy, n	Risk of bias
CCG-1942	Jacobs et al.[48]	2005	Conference abstract	Prospective cohort	Not reported	Not reported	58	4 (1–10)	47	ALL	Not reported	See S4 Appendix
	Jacobs et al.[11]	2007	Original article	Prospective cohort	Multi-centre, USA	1/1995–4/1996	58	4 (1–10)	47	ALL	MTX; prednisone; VCR	
CCG-1952	Lowe et al.[49]*	2001	Original article	Prospective cohort	Multi-centre, USA	1/1995–4/1996	41	3 (1–9)	51	ALL	MTX; prednisone; VCR	
	Wray et al.[12]	2014	Original article	Retrospective cohort	Multi-centre, USA	1996–2000	351	1–10	54	ALL	Not reported	
	Cheung et al.[50]	2003	Original article	Prospective cohort	Single-centre, Australia	Not reported	15	34 (17–65)	40	IBD (CD 13, UC 1, IC 1)	6MP, 5; AZA, 1; entocort, 1; prednisone, 6	Fair
Deibert et al. 2003	Deibert et al.[8]*	2003	Original article	Cross-over study	Germany	Not reported	6	44 (27–54)	33	IBD (CD)	Steroids	Fair
	Deibert et al.[51]*	2003	Conference abstract	Cross-over study	Not reported	Not reported	6	42 mean (SD 12)	33	IBD (CD)	Steroids	
Dubinsky et al. 2003	Dubinsky et al.[52]	2000	Conference abstract	Prospective cohort	Not reported	Not reported	8	Not reported	Not reported	IBD (CD 6, UC 2)	Not reported	Fair
	Dubinsky et al.[53]	2001	Conference abstract	Prospective cohort	Not reported	Not reported	17	43 (9–63)	Not reported	IBD	Not reported	
	Dubinsky et al.[54]	2001	Original article	Prospective cohort	Single-centre, USA	7/1999–7/2002	10	40 (9–64)	Not reported	IBD (CD)	Steorids, 6	
	Dubinsky et al. [17]	2003	Original article	Prospective cohort	Single-centre, USA	Not reported	21	38 (24–63)	57	IBD (CD 14, UC 7)	Not reported	
	Dubinsky et al. [55]	2003	Original article	Prospective cohort	Single-centre, USA	11/1999–2/2002	111, group 1, with laboratory abnormalities† (n=29); group 2, no laboratory abnormalities (n=82)	Group 1, 42 (17–70); group 2, 38 (9–81)	52	IBD (CD 77, UC 30, IC 3)	INF, 6; mesalamine 67; prednisone, 46	
	Dubinsky et al.[56]	2003	Conference abstract	Prospective cohort	Not reported	Not reported	111	Not reported	Not reported	IBD	Not reported	
	Geller et al.[57]	2003	Conference abstract	Retrospective cohort	Single-centre, USA	Not reported	125	Not reported	Not reported	IBD	Not reported	
	Geller et al.[58]	2004	Original article	Retrospective cohort	Single-centre, USA	Not reported	111	39 (9–67)	68	IBD	Not reported	
	Poordad et al.[16]	2002	Conference abstract	Retrospective cohort	Single-centre, USA	1999–2002	125	40	65	IBD	Not reported	
Ferlitsch et al. 2007	Ferlitsch et al.[59]	2005	Conference abstract	Prospective cohort	Not reported	Not reported	24	39 (23–76)	46	IBD (CD 21, UC 2, IC 1)	Not reported	Fair
	Ferlitsch et al.[18]	2007	Original article	Prospective cohort	Single-centre, Austria	11/2004–7/2005	26	41 (23–76)	42	IBD (CD 23, UC 2, IC 1)	5-ASA, 5; INF, 3; investigational biologicals, 2; steroids, 5	

	Hasyagar et al.[60]	2007	Conference abstract	Retrospective cohort	Single-centre, USA	2002–2007	24	48 mean (17–86)	29	IBD (CD 23, UC 1)	Not reported	n/a
	Ivastinovic et al.[61]	2004	Conference abstract	Prospective cohort	Not reported	Not reported	12	20–46	Not reported	IBD (CD)	INF, 4; prednisone, 2	n/a
Jharap et al. 2011	Jharap et al.[62]*	2008	Conference abstract	Cross-over study	Single-centre	Not reported	12	44 mean (26–61)	17	IBD (CD 6)	Not reported	Fair
	Jharap et al.[10]*	2011	Original article	Cross-over study	Single-centre, Netherlands	7/2005 – 6/2007	12	41 (26–61)	17	IBD (CD 6, UC 6)	Mesalazine 5	
	Kornbluth et al.[63]	2002	Conference abstract	Prospective cohort	USA	Not reported	15	Not reported	Not reported	IBD (CD 10, UC 5)	Steroids	n/a
	Lancaster et al.[7]*	2001	Original article	Cross-over study	Single-centre, UK	Not reported	11	4 (2–7)	Not reported	ALL	Cytarabine, daunorubicin, etoposide, steroids, VCR	n/a
	Omer et al.[64]	2015	Conference abstract	Retrospective cohort	Single-centre, UK	2012–2015	28	44 mean (19–67)	50	IBD (CD 13, UC 14, IC 1)	Not reported	n/a
Pavlidis et al. 2014	Pavlidis et al.[65]	2014	Conference abstract	Retrospective cohort	Multi-centre, UK, Australia	Not reported	62	Not reported	Not reported	IBD (CD 21, UC/IC 41)	Biologic therapy, calcineurin inhibitor therapy, steroids	Fair
	Pavlidis et al.[20]s	2014	Original article	Retrospective cohort	Multi-centre, UK, Australia	2009–7/2013	62	42 (20–75)	40	IBD (CD 21, UC 41)	5-ASA, 32; anti-TNF, 9; calcineurin inhibitor, 15; cyclosporine or tacrolimus, 5	
	Qasim et al.[66]	2007	Original article	Prospective cohort	Single-centre, Ireland	2/2002–12/2003	40	33 (18–69)	43	IBD (CD 28, UC 10, IC 2)	5-ASA, 36; steroids, 34	Fair
	Saleem et al.[67]	2013	Conference abstract	Retrospective cohort	Single-centre, UK	Not reported	12	37 mean (19–62)	42	IBD (CD 6, UC 6)	Not reported	n/a
Teml et al. 2007	Almer et al.[68]	2007	Conference abstract	Prospective cohort	Single-centre, Sweden	Not reported	23	41 (19–65)	57	IBD (CD)	Not reported	Fair
	Almer et al.[69]	2009	Original article	Prospective cohort	Single-centre, Sweden	12/2000–1/2004	23	41 (19–65)	57	IBD (CD)	5-ASA 8; INF, 1; steroids, 15	
	Bernstein et al.[70]	2002	Conference abstract	Retrospective cohort	Single-centre	Not reported	28	Not reported	57	IBD (CD 22, UC 6)	Not reported	n/a
	Bokemeyer et al.[71]	2003	Original article	Prospective cohort	Single-centre, Germany	Not reported	11	Not reported	Not reported	IBD	INF, tacrolimus	Fair
	Bonaz et al.[72]	2002	Conference abstract	Prospective cohort	Not reported	Not reported	28	32	14	IBD (CD)	Not reported	Fair
	Bonaz et al. [73]	2003	Original article	Prospective cohort	Multi-centre, France/ Belgium	12/2000–2/2002	49	32 (19–55)	27	IBD (CD)	Budesonide, 5; INF, 11; prednisone 21	
	Herrlinger et al.[15]	2002	Conference abstract	Prospective cohort	Germany	Not reported	35	Not reported	Not reported	IBD (CD)	Not reported	Fair
	Herrlinger et al.[74]	2003	Original article	Prospective cohort	Germany	Not reported	16	35 (22–58)	31	IBD (CD)	Prednisone, 6	
	Herrlinger et al.[75]	2003	Conference abstract	Prospective cohort	Germany	Not reported	16	Not reported	Not reported	IBD (CD)	Not reported	
	Herrlinger et al.[76]	2003	Original article	Prospective cohort	Multi-centre, Germany	Not reported	37	36 (22–61)	38	IBD (CD)	5-ASA; steroids, 27	
	Herrlinger et al.[77]	2004	Original article	Prospective cohort	Germany	Not reported	26	35 (22–57)	0	IBD (CD)	5-ASA; steroids	

	Klugman et al.[78]	2005	Original article	Retrospective cohort	Not reported	2001	23	Not reported	Not reported	IBD (CD 19, UC 4)	Not reported	Fair
	Seiderer et al.[79]	2004	Conference abstract	Prospective cohort	Multi-centre	Not reported	22	18–58	Not reported	IBD (CD 15, UC 7)	Not reported	Fair
	Seiderer et al.[80]	2005	Original article	Prospective cohort	Multi-centre, Germany, Czech Republic, Austria	Not reported	45	37 (20–73)	44	IBD (CD 32, UC 11, IC 2)	Steroids, 15	
	Teml et al.[81]	2003	Conference abstract	Prospective cohort	Not reported	Not reported	20	Not reported	Not reported	IBD (UC 14, IC 6)	Steroids	Poor
	Teml et al.[82]	2005	Original article	Prospective cohort	Multi-centre, Germany, Austria	8/2001–3/2002	20	45 (19–73)	35	IBD (UC 14, IC 6)	5-ASA, 6; cyclosporine, 2; INF, 2; steroids, 15	
	Teml et al.[83]	2005	Conference abstract	Retrospective cohort	Not reported	Not reported	69	Not reported	Not reported	IBD (CD 50, UC 14, IC 5)	Steroids, 49	
	Teml et al.[19]	2007	Original article	Prospective cohort	Multi-centre, Germany, Austria, Netherlands, Sweden, Czech Republic, France, Australia	11/2001–12/2004	296	37 (16–82)	40	IBD (CD 239, UC 48, IC 9)	5-ASA, 95; INF, 65; steroids, 171	
	Zech et al.[84]	2007	Original article	Prospective cohort	Multi-centre, Germany, Austria	Not reported	31	40 mean (20–73)	39	IBD (CD 19, UC 10, IC 2)	Not reported	Fair
	Harrer et al.[85]	2003	Conference abstract	Prospective cohort	Not reported	Not reported	45	Not reported	Not reported	IBD (CD)	Not reported	See Teml et al.
	Thomson et al.[14]	2004	Original article	Prospective cohort	Multi-centre, USA	5/1997–5/2001	30	5 (1–16)	57	ALL	MTX, VCR	Fair
UK MRC ALL 97 & 97/99	Piel et al.[86]	2004	Letter to the editor	Retrospective cohort	Single center, UK	2002	59	Not reported	57	ALL	Not reported	See S4 Appendix
	Ramanujachar et al.[87]	2007	Original article	Retrospective cohort	Multi-centre, UK	1997–2002	128	16 (15–17)	62	ALL	MTX, steroid (randomised: prednisoone/dexamethasone), VCR	
	Ravikumara et al.[88]	2005	Conference abstract	Retrospective cohort	Multi-centre, UK	10/1997–3/2004	9	11 (5–15)	78	ALL	Not reported	
	Ravikumara et al.[89]	2006	Original article	Retrospective cohort	Multi-centre, UK	12/2001–2/2005	75	11 (6-15)	70	ALL	Not reported	
	Roy Moulik et al.[90]	2015	Conference abstract	Retrospective cohort	Single-centre, UK	Not reported	59	Not reported	73	ALL	Not reported	
	Roy Moulik et al.[13]	2017	Original article	Retrospective cohort	Single-centre, UK	Not reported	59	7 (2–14)	73	ALL	Not reported	
Van Asseldonk et al. 2016	De Boer et al.[91]	2005	Original article	Retrospective cohort	Single-centre, Netherlands	8/2002–11/2004	25	Female, 40 mean (SD 12); male, 42 mean (SD 13)	44	IBD (CD 12, UC 5)	None	Fair
	De Boer et al.[92]	2005	Original article	Retrospective cohort	Multi-centre, Netherlands	6/2001–7/2003	95	43 (mean, 20-74)	39	IBD (CD 53, UC	None	

										42)		
	De Boer et al.[93]	2005	Conference abstract	Retrospective cohort	Not reported	Not reported	95	Not reported	Not reported	IBD	Not reported	
	De Boer et al.[94]*	2007	Original article	Prospective cohort	Netherlands	Not reported	7	42 (20–54)	14	IBD (CD)	None	
	De Boer et al.[95]	2007	Conference abstract	Prospective cohort	Netherlands	Not reported	30	Not reported	57	IBD (CD 17, UC 13)	Not reported	
	De Boer et al.[96]	2008	Original article	Prospective cohort	Multi-centre, Netherlands	2001–2002	29	44 (22–68)	61	IBD (CD 17, UC 11)	INF, 6; mesalazine, 16; prednisone, 11	
	De Jong et al.[97]	2002	Conference abstract	Prospective cohort	Netherlands	Not reported	31	Not reported	Not reported	IBD	Not reported	Fair
	Derijks et al.[98]	2002	Conference abstract	Prospective cohort	Netherlands	Not reported	9	41 mean (24–59)	33	IBD (CD 5, UC 4)	Not reported	
	Derijks et al.[99]	2003	Original article	Prospective cohort	Multi-centre, Netherlands	Not reported	32	37 mean (19–64)	22	IBD (CD 22, UC 10)	None	
	Derijks et al.[100]*	2006	Original article	Prospective cohort	Multi-centre, Netherlands	5/2001–11/2003	28	38 mean (19–70)	21	IBD (CD 16, UC 12)	5-ASA, 23; steroids	
	Gilissen et al.[101]	2007	Original article	Prospective cohort	Single-centre, Netherlands	Not reported	13	41 (28–53)	23	IBD (CD 8, UC 5)	Not reported	
	Jharap et al.[102]	2007	Conference abstract	Retrospective cohort	Multi-centre, Netherlands	Not reported	73	45 (22–72)	47	IBD (68)	Not reported	n/a
	Van Asseldonk et al.[103]	2009	Conference abstract	Retrospective cohort	Multi-centre, Netherlands	Not reported	122	Not reported	Not reported	IBD (CD 65, UC 57)	Not reported	Fair
	Van Asseldonk et al.[104]	2009	Conference abstract	Retrospective cohort	Multi-centre, Netherlands	Not reported	113	38 (16–62)	37	IBD (CD 62, UC 51)	Not reported	
	Van Asseldonk et al.[9]*	2010	Original article	Cross-over study	Netherlands	Not reported	18	45 mean (SD 11)	17	IBD (CD 6, UC 12)	Not reported	
	Van Asseldonk et al.[105]	2010	Conference abstract	Prospective cohort	Multi-centre, Netherlands	Not reported	99	44 mean (SD 12)	36	IBD (CD 61, UC 38)	Not reported	
	Van Asseldonk et al.[106]	2010	Conference abstract	Retrospective cohort	Not reported	Not reported	11	50 mean (SD 13)	27	IBD (CD 5, UC 6)	Not reported	
	Van Asseldonk et al.[107]	2011	Original article	Retrospective cohort	Multi-centre, Netherlands	2001–2007	46	38 (19–71)	50	IBD (UC)	5-ASA, 36; CYC, 7; INF, 7; steroids, 38; tacrolimus, 1	
	Van Asseldonk et al.[108]	2012	Original article	Retrospective cohort	Single-centre, Netherlands	1/2006–7/2010	19	Mean 44 (SD 11)	26	IBD (CD 7, UC 12)	5-ASA, 8; adalimumab, 1; steroids, 7	
	Van Asseldonk et al.[109]	2014	Conference abstract	Retrospective cohort	Multi-centre, Netherlands	Not reported	23	Not reported	39	IBD	Not reported	
	Van Asseldonk et al.[21]	2016	Original article	Retrospective cohort	Multi-centre, Netherlands	2002–2014	111	44 (SD 13)	38	IBD (CD 69, UC 42)	Adalimumab, 2; CYC, 1; INF, 18; steroids, 40; tacrolimus: 4	
Ward et al. 2017	Ansari et al.[110]	2008	Original article	Retrospective cohort	Single-centre, UK	2001–2006	30	34 (12–57)	40	IBD (CD)	Steroids	Fair

	Elliott et al.[111]	2007	Conference abstract	Retrospective cohort	Not reported	2001–2006	29	Not reported	Not reported	IBD (CD)	Steroids	
	Patel et al.[112]	2013	Conference abstract	Retrospective cohort	Single-centre, UK	2008–2012	37	43 mean	41	IBD (CD 29, UC 6, IC 1)	Adalimumab, 1	
	Soon et al.[113]	2004	Conference abstract	Prospective cohort	Single-centre, UK	Not reported	25	Not reported	Not reported	IBD (CD)	INF, steroids	
	Ward et al.[114]	2013	Conference abstract	Retrospective cohort	Single-centre, UK	1/2001–1/2013	55	Not reported	45	IBD (CD 46)	Not reported	
	Ward et al.[22]	2017	Original article	Retrospective cohort	Single-centre, UK	2001–2013	54	35 (IQR 27-49)	35	IBD (CD 47, UC 7)	Anti-TNF, 18	

5-ASA; 5-amino salicylic acid; AZA, azathioprine; ALL, acute lymphoblastic leukaemia; CD, Crohn's disease; CIVI, continuous intravenous infusion; IBD, inflammatory bowel disease; INF, infliximab; IC, indeterminate colitis; MTX, methotrexate; n/a, not applicable; UC, ulcerative colitis; VCR, vincristine; TNF, tumour necrosis factor.

* Studies only concerning pharmacokinetics

† Abnormal liver chemistry and/or evidence of haematologic toxicity attributable to 6TG therapy.[17]

Table C. Study characteristics of included case reports

Study	Report	Year	Setting	Number of patients	Age, years median (range)	Gender, % male	Disease	Concomitant therapy	Risk of bias
	Bisschop et al.[115]	2001	Single center, France	1	10	0	ALL	Not reported	n/a
CCG-1952	Broxson et al.[116]	2001	Single center, USA	12	1.5–7.25	Not reported	ALL	MTX, prednisone, VCR	See S4 Appendix
	Broxson et al.[25]	2005	Single center, USA	12	6 (3–10)	67	ALL	MTX, prednisone, VCR	
	Chojnacki et al.[117]	2012	Poland	1	14	0	IBD	5-ASA, prednisone	n/a
	De Boer et al.[118]	2005	Single center, Netherlands	8	Adult	50	IBD	Not reported	Fair
UK MRC ALL 97/99	De Bruyne et al.[23]	2006	Single center, UK	6	6.6 (3.25–11.5)	0.8	ALL	Steroid, VCR, MTX	See S4 Appendix
	Rawat et al.[24]	2011	Multicenter, UK	10	17 (13–22)	0.7	ALL	Steroid, VCR, MTX	
	Fritz et al.[119]	2008	Single center, Austria	1	56	0	IBD	Prednisone 12.5 mg/day	Fair
	Kane et al.[120]	2004	Single center, USA	1	40	100	IBD	None	Fair
	Mao et al.[121]	2013	China	2	6–7	50	ALL	VTLD, CAT, HDMTX	n/a
	Marasco et al.[122]	2016	Single center, Italy	1	50	100	IBD	Not reported	Fair
	Mares et al.[123]	2009	Single center, Netherlands	1	34	100	IBD	Infliximab	Poor
	Wong et al.[124]	2009	Single center	1	33	100	IBD	Infliximab 5 mg/kg every 8 weeks	
	Merino et al.[125]	2000	Single center, Spain	1	4	100	ALL	MTX (weekly), silybum marianum (herbal remedy)	Fair
	Nielsen et al.[126]	2015	Single center, Denmark	1	8	0	ALL	MTX, 6MP, VCR, dexamethasone, cytarabine, prednisone	Fair
	Radys et al.[127]	2006	Poland	1	14	0	IBD	None	n/a
	Rulyak et al.[128]	2003	Single center, USA	1	27	0	IBD	Prednisone 40 mg/d	Poor
	Salerno et al.[129]	2014	Single center, Italy	1	44	100	IBD	None	Fair
Dubinsky et al. 2003	Shastri et al.[130]	2002	Single center, USA	3	Not reported	Not reported	IBD	Not reported	See Table B
	Shastri et al.[131]	2004	USA	3	36 (33–39)	100	IBD	5-ASA n=1	
Van den Berg et al. 2016	De Boer et al.[132]	2005	Netherlands	2	23–24	0	IBD	Case 1: none, case 2: prednisone 40 mg/d	Fair
	Van den Berg et al.[133]	2016	Multicentre, Netherlands	13	20 (IQR 17–22)	0	IBD	Anti-TNF-a n=2, 5-ASA n=2, steroid n=4	
Ivastinovic et al. 2004[61]	Wenzl et al.[134]	2004	Single center, Austria	1	36	0	IBD	Not reported	See Table B

5-ASA, 5-aminosalicylic acid; 6-MP, 6-mercaptopurine; ALL, acute lymphoblastic leukaemia; HDMTX, high-dose methotrexate; IBD, inflammatory bowel disease; MTX, methotrexate; VCR, vincristine.

Table D. Results of randomised controlled trials

Trial	Report	Year	Duration of 6TG (months), median (range)	Dose of 6TG (mg/m^2/d), median (range)	Ery-TGN pmol/8·10^8 RBC, median (range)	Incidence of hepatotoxicity 6TG group in MT, n (%)	Follow-up (years), median (range)	Biopsy, n (% of 6TG group)	Other diagnostic methods, n (% of 6TG group)	Truncation of 6TG due to hepatotoxicity, n (% of 6TG group)
ALL-REZ BFM 87	Einsiedel et al.[1]	2005	Group A+B, 24; group C, 12	50	Not reported	Not reported	15	Not reported	Not reported	Not reported
CCG-1952	Matloub et al.[26]	2006	Girls 26, boys 38	50–60	Not reported	Not reported	6.0	Not reported	Not reported	Not reported
	Stork et al.[27]	1998		50–60	Not reported	(6)	Not reported	5	Doppler US, 20	Not reported
	Stork et al.[28]	2002		50–60	Not reported	200 (20)	Not reported	Not reported	Not reported	200 (20)
	Stork et al.[5]	2002	22 (mean)	50–60	Patients with splenomegaly (n=6), 326 (mean, range 144-464); other patients on TG (n=20), 306 (mean, range 104-1021); patients on MP (n=20), 53 (mean, range 27-113)	44 (4)	Not reported	12 (1)	Doppler US, MRI/MRA and/or upper GI endoscopy, 13 (1)	Not reported
	Stork et al.[2]	2010	Girls 24, boys 36 from first IM	50–60	Not reported	257 (25)	7.0	8 (1)	Doppler US, 168 (17)	257 (25)
CO ALL-92	Erb et al.[29]*	1998	18	50–100	2538 (511–5384)	Not reported	2.3	Not reported	Not reported	Not reported
	Harms et al.[30]	2000	Not reported	Not reported	Not reported	Not reported	4.9 (2.1–7.6)	Not reported	Not reported	Not reported
	Harms et al.[3]	2003	24	36, mean	Not reported	0	6.6	Not reported	Not reported	Not reported
UK MRC ALL 97/ Pilot	Lancaster et al.[31]	1998	12 (7–19)	4, mean (2–9)	2168 (790–7090)	0	Not reported	Not reported	Not reported	Not reported
	Stoneham et al.[6]	2003	>12	34–35	SOS, 1592 (1240–1965); no SOS, 1647 (1231–1979)	12 (19)	Not reported	1 (2)	Doppler US, 10 (16)	10 (16)
UK MRC ALL97 & UKALL XI	Hann et al.[32]	2000	23	40	Not reported	Not reported	4.0 (0.4–8.0)	Not reported	Not reported	Not reported
UK MRC ALL 97 & 97/99	Cartwright et al.[33]	2008	Girls 24, boys 36 from first IM	40	1734 (759–4002)	Not reported	6.1 (3.5–9.7)	Not reported	Not reported	Not reported
	Cartwright et al.[34]*	2011		40	Not reported	Not reported	Not reported	Not reported	Not reported	Not reported
	Lancaster et al.[35]*	2002		43 (36–58)	1472 (1045–2459)	Not reported	Not reported	Not reported	Not reported	Not reported
	Lennard et al.[36]	2001		40	1372 (659–2459)	Not reported	Not reported	Not reported	Not reported	Not reported

	Lennard et al.[37]	2003		40 (34–60)	1952 (1162–5414)	65 (9)	2.8	Not reported	Not reported	Not reported
	Lennard et al.[38]	2005		40	Not reported	82 (11)	Not reported	Not reported	Not reported	Not reported
	Lennard et al.[39]	2006		40	SOS, 2034 (682–4072); control on TG, 1916 (718–3786)	83 (11)	4.6	Not reported	Not reported	83 (11)
	Lennard et al.[40]	2008		40 (10–78)	1940 (36–6874)	Not reported	Not reported	Not reported	Not reported	Not reported
	Lennard et al.[41]	2008		40 (10–78)	1940 (36–6874)	Not reported	Not reported	Not reported	Not reported	Not reported
	Lennard et al.[42]*	2013		40 (10–78)	1940 (36–6874)	Not reported	Not reported	Not reported	Not reported	Not reported
	Lennard et al.[43]*	2015		40 (14–78)	WT TPMT, 1904 (36–4336); heterozygenous TPMT, 2468 (174–6730)	Not reported	Not reported	Not reported	Not reported	Not reported
	Mitchell et al.[44]	2002		Not reported	Not reported	Not reported	2.3	Not reported	Not reported	Not reported
	Mitchell et al.[45]	2009		40	Not reported	Not reported	8.0 (5.0–11.0)	Not reported	Not reported	Not reported
	Mitchell et al.[46]	2010		40	Not reported	85 (11)	6.0–11.0	Not reported	Not reported	Not reported
	Vora et al.[47]	2002		40 (34–60)	40 mg/m^2, 1860 (n=225); 50 mg/m^2, 2204 (n=72)	40 (6)	2.3	Not reported	Not reported	Not reported
	Vora et al.[4]	2006		40	Not reported	68 (9)	6.0	10 (1)	Upper GI endoscopy, 12 (2)	68 (9)

6TG, 6-thioguanine; GI, gastro intestinal; IM, interim maintenance; MRA, magnetic resonance angiography; MRI, magnetic resonance imaging; MT, maintenance; PY, patient year; US, ultrasound.

Doses in mg/m^2 were calculated with the assumption that an adult is 1.73 m^2

* Studies only concerning pharmacokinetics or TPMT activity

Table E. Results of observational studies

Study	Report	Year	Duration of 6TG (months), median (range)	Dose of 6TG (mg/m2/day), median (range)	Cumulative dose of 6TG (g), median (range)	Ery-TGN pmol/8*10^8 RBC, median (range)	Incidence of hepatotoxicity, n (%)	Follow-up, median (range)	Biopsy, n (% of total)	Other diagnostic methods than biopsy + n (% of total)	Dose reduction of 6TG due to hepatotoxicity, n (% of total)	Truncation of 6TG due to hepatotoxicity, n (% of total)
CCG-1942	Jacobs et al.[48]	2005	Not reported	60–480 (oral, 60; CIVI; 480 over 24 hours every 8 weeks of first year of MT)	Not reported	570 (72–2682)	6 (10)	9 years	Not reported	Not reported	Not reported	6 (10)
	Jacobs et al.[11]	2007	17 (12–26)	60–480 (oral, 60; CIVI; 480 over 24 hours every 8 weeks of first year of MT)	Not reported	2 weeks after MT cycle 1, 432 (72–1920); 2 weeks after MT cycle 2, 492 (132–2682); end of CIVI, 3906 (1980–7644)	30 (52)	9 (2–9) years	Not reported	US, 6 (10)	Not reported	6 (10)
CCG-1952	Lowe et al.[49]*	2001	36	60–480 (oral, 60; CIVI; 480 over 24 hours every 8 weeks of first year of MT)	Not reported	7 days after CIVI, 1344 (SD 600); after daily oral 6TG, 643 (SD 492)	Not reported	Not reported	Not reported	Not reported	Not reported	Not reported
	Wray et al.[12]	2014	Not reported	50–60	Not reported	Not reported	79 (23)	Not reported	Not reported	Not reported	Not reported	Not reported
	Cheung et al.[50]	2003	4 (1–5)	23	Not reported	Not reported	0	16 (3–21) weeks	Not reported	Not reported	Not reported	Not reported
Deibert et al. 2003	Deibert et al.[8]*	2003	Three single doses of 6TG with wash-out periods of at least 7 days	29 mean (SD 0.6)	Not reported	Not reported	Not reported	Not reported	Not reported	Not reported	Not reported	Not reported
	Deibert et al.[51]*	2003	Three single doses of 6TG	23	Not reported	Not reported	Not reported	Not reported	Not reported	Not reported	Not reported	Not reported
Dubinsky et al. 2003	Dubinsky et al.[52]	2000	4	23	Not reported	Week 2, 743; week 4, 1045; week 16, 781	0	16 weeks	Not reported	Not reported	Not reported	Not reported
	Dubinsky et al.[53]	2001	Not reported	12 (6–23)	Not reported	Not reported	0	2 weeks	Not reported	Not reported	0	Not reported
	Dubinsky et al.[54]	2001	4	23 (12–58)	Not reported	1388 (275–2073)	0	16 weeks	Not reported	Not reported	0	0
	Dubinsky et al. [17]	2003	9 (1–24)	12 (6–23)	Not reported	1200 (680–2964)	0	Not reported	Not reported	Not reported	0	0
	Dubinsky et al. [55]	2003	Group 1, 12 (1–26); group 2, 9 (1–28)	Not reported	Group 1, 16 (1–34); group 2, 12 (1–36) mg	Group 1, 1230 (502–2310); group 2, 1280 (510–2881)	27 (24)	Not reported	26 (23) (17 from group 1, 9 from group 2)	Not reported	Not reported	Not reported
	Dubinsky et al.[56]	2003	Not reported	Not reported	Not reported	Not reported	27 (24)	Not reported	17 (15)	Not reported	Not reported	Not reported

	Geller et al.[57]	2003	12–36	Not reported	Not reported	Not reported	16 (13)	Not reported	32 (26)	EM, 11 (9)	Not reported	Not reported
	Geller et al.[58]	2004	12–36	Not reported	Not reported	Not reported	Not reported	Not reported	38 (34)	EM, 23 (21)	Not reported	Not reported
	Poordad et al.[16]	2002	NRH, 15 (5–24); No NRH, 11 (2–17)	Not reported	NRH, 24 (3–34); No NRH, 23 (4–32)	NRH, 1323 (786–2150); No NRH, 1344 (736–2017)	14 (11)	Not reported	20 (16)	Not reported	Not reported	n/a
Ferlitsch et al. 2007	Ferlitsch et al.[59]	2005	38 (12–41)	At time of HVPG, 6 (3–23); max, 23 (12–46)	Not reported	Not reported	5 (23)	Not reported	22 (92)	HVPG, 24 (100)	Not reported	5 (23)
	Ferlitsch et al.[18]	2007	38 (12–45)	At time of HVPG, 6 (3–23); max, 23 (12–46)	27 (7–56)	Not reported	6 (23)	1 year	24 (92)	HVPG, 26 (100)	Not reported	6 (23)
	Hasyagar et al.[60]	2007	21 mean (2–72)	Not reported	Not reported	Not reported	2 (8)	Not reported	2 (8)	Not reported	Not reported	1 (4)
	Ivastinovic et al.[61]	2004	12 (6–27)	23	Not reported	774 (244–1404)	1 (8)	12 (6–27) months	1 (8)	Not reported	Not reported	1 (8)
Jharap et al. 2011	Jharap et al.[62]*	2008	Two doses with 7 day interval, one oral and one 30 minute IV infusion	0.3 mg/kg	Not reported	Oral, 285 (30–563); IV, 902 (538–2275) (peak concentration)	Not reported	Not reported	Not reported	Not reported	Not reported	Not reported
	Jharap et al.[10]*	2011	Two doses with 7 day interval, one oral and one 30 minute IV infusion; 4 weeks in 8 patients	12 (10–14)	Not reported	Oral, 141 (30–563); IV, 902 (538–2006); steady state after 4 weeks, 1,215 (300–6070)	Not reported	Not reported	Not reported	Not reported	Not reported	Not reported
	Kornbluth et al.[63]	2002	Not reported	14 mean (6–34)	Not reported	689 mean (353–820)	Not reported	Not reported	Not reported	Not reported	Not reported	Not reported
	Lancaster et al.[7]*	2001	Two 4 week periods	40	Not reported	6 h (fasting), 295 (130–477); 6 h (food), 210 (103–544); steady state (fasting), 1742 (875–3026); steady state (food), 1600 (972–2894)	Not reported	Not reported	Not reported	Not reported	Not reported	Not reported
	Omer et al.[64]	2015	14 (1–40)	Not reported	Not reported	Not reported	0	Not reported	Not reported	MRI	Not reported	0
Pavlidis et al. 2014	Pavlidis et al.[65]	2014	8 (0–45)	Not reported	Not reported	Not reported	2 (3)	Not reported	9 (15)	MRI, 2; US, 21	1 (2)	1 (2)
	Pavlidis et al.[20]	2014	8 (0–45)	0.6 mg/kg/d (0.3–1.0)	Not reported	811 (340–2678)	2 (3)	10 (0–45)	11 (18)	MRI, 2 (3); US, 21 (34)	1 (2)	1 (2)
	Qasim et al.[66]	2007	8 (0–20)	23	Not reported	Not reported	8 (20)	34 (2–90) weeks	Not reported	US, 8 (20)	n/a	8 (20)
	Saleem et al.[67]	2013	8 (1–12)	12	Not reported	Not reported	0	6 months	Not reported	Not reported	None	None

Teml et al. 2007	Almer et al.[68]	2007	9 (1–48)	23 (12–35)	Not reported	Patients with AE (n=16), 652 (99-2488); patients without AE (n=7), 551 (392-1574)	2 (9)	Not reported	Not reported	Not reported	Not reported	2 (9)
	Almer et al.[69]	2009	9 (1–48)	23 (12–35)	Not reported	Patients with AE (n=16), 652 (99-2488); patients without AE (n=7), 551 (392-1574)	2 (9)	Not reported	2 (9)	CT, 3 (13); MR, 6 (26); US, 12 (52)	0	2 (9)
	Bernstein et al.[70]	2002	4 mean (1–9)	12	Not reported	546 (113–3114)	Not reported	Not reported	Not reported	Not reported	Not reported	Not reported
	Bokemeyer et al.[71]	2003	Not reported	23	Not reported	Not reported	Not reported	Not reported	Not reported	Not reported	Not reported	0
	Bonaz et al.[72]	2002	3 mean (0–5)	12	Not reported	750 mean (379–1240)	1 (4)	3 mean (0–5)	Not reported	Not reported	Not reported	1 (4)
	Bonaz et al. [73]	2003	7 (1–20)	12 (12–23)	Not reported	648 (154–1316)	2 (4)	7 (1–20) months	Not reported	Not reported	Not reported	2 (4)
	Herrlinger et al.[15]	2002	4	23 (23–46)	Not reported	Responders, 928 (SD 296); non-responders, 1005 (SD 428)	Not reported	16 weeks	Not reported	Not reported	Not reported	Not reported
	Herrlinger et al.[74]	2003	12	23 (12–46)	Not reported	1 month, 1265 (394–1853); 6 months, 1329 (865–2160); 12 months, 1067 (470–1631)	1 (6)	1 year	0	Doppler US, 1 (6)	Not reported	1 (6)
	Herrlinger et al.[75]	2003	12	12–23	Not reported	Not reported	1 (6)	1 year	Not reported	Not reported	Not reported	1 (6)
	Herrlinger et al.[76]	2003	5	23 (23–46)	Not reported	Not reported	2 (5)	24 weeks	Not reported	Not reported	0	0
	Herrlinger et al.[77]	2004	5	23 (23–46)	Not reported	4 weeks, 1241 (313–1853); 12 weeks, 956 (430–2668)	0	24 weeks	Not reported	Not reported	Not reported	0
	Klugman et al.[78]	2005	23 mean (1–36)	12–23	16 mean (1–37)	40 mg/d, 800-1200; 20 mg/d, 337 mean (88-602)	5 (22)	Not reported	5 (22)	MRI, 13 (57); US control every three months	Not reported	2 (9)
	Seiderer et al.[79]	2004	>6	6–23	Not reported	Not reported	5 (23)	Not reported	22 (100)	MRI, 22 (100)	Not reported	Not reported
	Seiderer et al.[80]	2005	16 (2–26)	23–46	16 (3–52)	Not reported	16 (36)	Not reported	45 (100)	MRI, 31 (69)	Not reported	Not reported
	Teml et al.[81]	2003	6	12–23	Not reported	Not reported	4 (20)	26 weeks	Not reported	Not reported	Not reported	Not reported
	Teml et al.[82]	2005	6	23 (12–46)	Not reported	816 (279–2300)	5 (31)	Not reported	1 (6)	Not reported	Not reported	1 (6)
	Teml et al.[83]	2005	24	23 (12–46)	Not reported	Not reported	Not reported	Not reported	Not reported	Not reported	Not reported	Not reported

	Teml et al.[19]	2007	12 (0–46)	12 (12–23) after 93 weeks, starting dose 23	Not reported	Not reported	43 (15)	370 PY	60 (20)	CT, 16 (5); MRI, 53 (18); US, 149 (50)	12 (4)	10 (3)
	Zech et al.[84]	2007	16 (5–26)	Not reported	18 mean (7–52)	Not reported	13 (42)	Not reported	31 (100)	MRI, 31 (100)	Not reported	Not reported
	Harrer et al.[85]	2003	6	23 (12–23)	Not reported	Not reported	Not reported	Not reported	Not reported	Not reported	Not reported	Not reported
	Thomson et al.[14]	2004	3	50	Not reported	Not reported	0	4 years	Not reported	Not reported	Not reported	Not reported
UK MRC ALL 97 & 97/99	Piel et al.[86]	2004	Not reported	Not reported	Not reported	Not reported	7 (12)	31 (18–54) months	4 (7)	US, 4 (7)	None	6 (10)
	Ravikumara et al.[88]	2005	Not reported	Not reported	Not reported	Not reported	9 (100)	26 (7–37) months	8 (89)	Upper GI endoscopy, 9 (100); US, 9 (100)	Not reported	Not reported
	Ravikumara et al.[89]	2006	Boys, 27–31; girls, 16–20	40	Not reported	Not reported	10 (13)	30 (5–46) months	9 (90)	Upper GI endoscopy, 10 (100); US, 10 (100)	Not reported	Not reported
	Roy Moulik et al.[90]	2015	Not reported	Not reported	Not reported	Not reported	11 (19)	132 (72–168) months	4 (36)	Upper GI endoscopy, 5 (45); US, 8 (73)	Not reported	Not reported
	Roy Moulik et al.[13]	2017	28 weeks (2-150)	Not reported	Not reported	Not reported	11 (19)	107 PY	4 (36)	Upper GI endoscopy, 6 (55); US, 8 (73)	Not reported	6 (10)
	Ramanujachar et al.[87]	2007	24–36	Not reported	Not reported	Not reported	Not reported	Not reported	Not reported	Not reported	n/a	n/a
Van Asseldonk et al. 2016	De Boer et al.[91]	2005	5.2 mean (SD 3.7)	12 (6–23)	Not reported	621 mean (34–1653)	Not reported	Not reported	Not reported	Not reported	Not reported	Not reported
	De Boer et al.[92]	2005	>12	14 (12–23)	Not reported	Patients with AE, 725 mean (229–1563); patients without AE, 540 mean (0–1404)	11 (12)	1 year	Not reported	US 51 (54)	Not reported	4 (4)
	De Boer et al.[93]	2005	Not reported	14	Not reported	540 (mean)	10 (11)	Not reported	1 (1)	US 51 (54)	Not reported	3 (3)
	De Boer et al.[94]*	2007	>1	12	Not reported	563 (158–858)	Not reported	Not reported	Not reported	Not reported	Not reported	Not reported
	De Boer et al.[95]	2007	39 mean (30–53)	12	23 (mean 12–44)	580 mean (106–1199)	1 (3)	>30 months	30 (100)	US, 30 (100)	Not reported	Not reported
	De Boer et al.[96]	2008	38 mean (30–53)	12 (3–23)	22 (SD 6)	564 (mean, SD 278)	0	38 months (30–53 months) from initition of 6TG to liver biopsy	28 (100)	Not reported	0	Not reported
	De Jong et al.[97]	2002	0–3	12 (12–23)	Not reported	20 mg/day, 790 (SD 374); 40 mg/day, 1393 (SD 752)	1 (3)	12 weeks	Not reported	Not reported	Not reported	1 (3)

	Derijks et al.[98]	2002	1	12 (12–23)	Not reported	986 (270–1666)	Not reported	4 weeks	Not reported	Not reported	Not reported	Not reported
	Derijks et al.[99]	2003	2	15 mean (6–23)	Not reported	20 mg/d, 937 mean (SD 325); 40 mg/d, 1621 mean (SD 828)	0	8 weeks	Not reported	Not reported	Not reported	Not reported
	Derijks et al.[100]*	2006	2	12	Not reported	848 mean (284–1888)	0	4–8 weeks	Not reported	Not reported	Not reported	Not reported
	Gilissen et al.[101]	2007	36 mean (27–45)	12 (4–12)	Not reported	802 (106–1092)	0	Not reported	13 (100)	MRI, 11 (85)	Not reported	Not reported
	Jharap et al.[102]	2007	34 (4–57)	12 (10–14)	Not reported	Not reported	Not reported	Not reported	73 (100)	Not reported	Not reported	Not reported
	Van Asseldonk et al.[103]	2009	40	0.3 mg/kg/d	Not reported	Not reported	Not reported	Not reported	Not reported	US	Not reported	Not reported
	Van Asseldonk et al.[104]	2009	26 (1–72)	0.3 mg/kg/d	Not reported	Not reported	1 (5)	245 PY	42 (37)	Not reported	Not reported	1 (1)
	Van Asseldonk et al.[9]*	2010	Two doses oral/IV 7 days apart	0.3 mg/kg	Not reported	Not reported	Not reported	Not reported	Not reported	Not reported	Not reported	Not reported
	Van Asseldonk et al.[105]	2010	25 (6–65)	0.28 (SD 0.07) mg/kg	Not reported	Not reported	4 (4)	Not reported	99 (100)	Not reported	Not reported	Not reported
	Van Asseldonk et al.[106]	2010	3 (1–18)	12 (6–14)	Not reported	1070 (390–2100)	2 (18)	3 (1–18)	Not reported	Not reported	Not reported	Not reported
	Van Asseldonk et al.[107]	2011	22 (0–73)	12 (10–14)	Not reported	278 (68–492)	2 (4)	22 (0–73)	12 (26)	US, 21 (46)	Not reported	2 (4)
	Van Asseldonk et al.[108]	2012	5 (1–21)	12 (SD 2)	Not reported	427 (126–800)	4 (21)	5 (1–21) months	2 (11)	Not reported	0	0
	Van Asseldonk et al.[109]	2014	26 (14–54)	12 (4–14)	Not reported	Not reported	0	26 (14–54)	23 (100)	US	Not reported	Not reported
	Van Asseldonk et al.[21]	2016	20 (7–64)	12 (SD 3)	13 (4–39)	464 (65–1199)	7 (6)	Not reported	111 (100)	CT, 2 (18); US, 76 (68)	Not reported	Not reported
Ward et al. 2017	Ansari et al.[110]	2008	22 (0–62)	23 (12–35)	23	807 (105–2545)	2 (7)	44 (3–62) months	11 (37)	MRI, 14 (47)	Not reported	2 (7)
	Elliott et al.[111]	2007	22 (0–60)	23 (12–34)	Not reported	Not reported	3 (10)	44 months	11 (38)	MRI and US, 20 (69)	Not reported	3 (10)
	Patel et al.[112]	2013	11 (IQR 3–30)	12–23	Not reported	1177 mean (70–3182)	1 (3)	Not reported	9 (24)	MRI, 9 (24)	None	1 (3)
	Soon et al.[113]	2004	6	12–23	Not reported	Not reported	1 (4)	Not reported	Not reported	Not reported	Not reported	1 (4)
	Ward et al.[114]	2013	14 (0–124)	23 (12–35)	10 (0–210)	848 (149–2789)	1 (2)	169 PY	Not reported	Not reported	Not reported	Not reported
	Ward et al.[22]	2017	16 (IQR 5–37)	16 mean (12–23)	13 (IQR 4–26)	740 (IQR 445–1078)	3 (6)	126 PY	11 (20)	MRI, 24 (44)	Not reported	2 (4)

6TG, 6-thioguanine; AE, adverse event; CIVI, continuous intravenous infusion; CSPH, clinical significant portal hypertension; CT, computed tomography; EM, electron microscopy; GI, gastro intestinal; HVPG, hepatic venous pressure gradient; IV, intra-venous; MRI, magnetic resonance imaging; MT, maintenance; PH, portal hypertension; PY, patient years; US, abdominal ultrasound.

Doses in mg/m^2 were calculated with the assumption that an adult is 1.73 m^2

Table F. Results of case reports

Study	Report	Year	Duration of 6TG (months), median (range)	Dose of 6TG (mg/m^2/day), median (range)	Incidence of hepatotoxicity, n (%)	Follow-up, median (range)	Biopsy, n (% of total)	Other diagnostic methods, n (% of total)	Truncation of 6TG due to hepatotoxicity, n (% of total)
	Bisschop et al.[115]	2001	Not reported	n/a	0	Not reported	Not reported	Not reported	0
CCG-1952	Broxson et al.[116]	2001	Not reported	34 (25–77)	12 (100)	Not reported	3 (25)	MRI, 9 (75); US, 7 (58)	Not reported
	Broxson et al.[25]	2005	20 (13–36)	34 (25–77)	12 (100)	7–44 months	3 (25)	MRI, 10 (83); Upper GI endoscopy, 4 (33); US, 5 (42)	7 (50)
	Chojnacki et al.[117]	2012	5	35	1 (100)	Not reported	0	Not reported	n/a
	De Boer et al.[118]	2005	21 (16–36)	12 (9–21)	0	Not reported	8 (100)	Not reported	Not reported
UK MRC ALL 97/99	De Bruyne et al.[23]	2006	12 (6–22)	40	6 (100)	23 (4–36) months	6 (100)	US, 6 (100); upper GI endoscopy, 3 (50)	6 (100)
	Rawat et al.[24]	2011	Not reported	n/a	10 (100)	65.7 PY	Not reported	Upper GI endoscopy 10 (100)	Not reported
	Fritz et al.[119]	2008	36	23	1 (100)	56 months	1 (100)	Not reported	1 (100)
	Kane et al.[120]	2004	13	23	1 (100)	Not reported	1 (100)	US, 1 (100); CT, 1 (100)	1 (100)
	Mao et al.[121]	2013	0–1	111–125*	2 (100)	Not reported	0	Not reported	Not reported
	Marasco et al.[122]	2016	Not reported	n/a	1 (100)	Not reported	0	MRI, 1 (100)	Not reported
Mares et al. 2009	Mares et al.[123]	2009	30	1–2	0	30 months	0	Not reported	0
	Wong et al.[124]	2009	Not reported	1–2	0	Not reported	Not reported	Not reported	0
	Merino et al.[125]	2000	4	75	1 (100)	8 months	1 (100)	Not reported	1 (100)
	Nielsen et al.[126]	2015	2	3–10	0	5.9 years	0	US, 1 (100)	0
	Radys et al.[127]	2006	1	n/a	1 (100)	Not reported	0	Not reported	Not reported
	Rulyak et al.[128]	2003	1	23	1 (100)	2 months	0	CT, 1 (100)	1 (100)
	Salerno et al.[129]	2014	3	14–46	1 (100)	3 months	1 (100)	US, 1 (100); CT, 1 (100)	1 (100)
Dubinsky et al. 2003	Shastri et al.[130]	2002	12	n/a	3 (100)	Not reported	3 (100)	Not reported	Not reported
	Shastri et al.[131]	2004	17 (15–18)	n/a	3 (100)	Not reported	3 (100)	Not reported	Not reported
Van den Berg et al. 2016	De Boer et al.[132]	2005	17–18	2–12	0	17–18 months	Not reported	Not reported	0
	Van den Berg et al.[133]	2016	33	5–10	0	Not reported	Not reported	Not reported	Not reported

Ivastinovic et al. 2004[61]	Wenzl et al.[134]	2004	14	23	1 (100)	7 weeks	0	US, 1 (100)	1 (100)

CT, computed tomography; MRI, magnetic resonance imaging; PY, patient years; US, abdominal ultrasound.

Doses in mg/m^2 were calculated with the assumption that an adult is 1.73 m^2

*Calculated using mean weight and height for a 6 and 7 year old child

References

1. Einsiedel HG, von Stackelberg A, Hartmann R, Fengler R, Schrappe M, Janka-Schaub G, et al. Long-term outcome in children with relapsed ALL by risk-stratified salvage therapy: results of trial acute lymphoblastic leukemia-relapse study of the Berlin-Frankfurt-Munster Group 87. J Clin Oncol. 2005;23(31):7942–50.
2. Stork LC, Matloub Y, Broxson E, La M, Yanofsky R, Sather H, et al. Oral 6-mercaptopurine versus oral 6-thioguanine and veno-occlusive disease in children with standard-risk acute lymphoblastic leukemia: report of the Children's Oncology Group CCG-1952 clinical trial. Blood. 2010 Apr 8;115(14):2740–8.
3. Harms DO, Gobel U, Spaar HJ, Graubner UB, Jorch N, Gutjahr P, et al. Thioguanine offers no advantage over mercaptopurine in maintenance treatment of childhood ALL: Results of the randomized trial COALL-92. Blood. 2003;102(8):2736–40.
4. Vora A, Mitchell CD, Lennard L, Eden TOB, Kinsey SE, Lilleyman J, et al. Toxicity and efficacy of 6-thioguanine versus 6-mercaptopurine in childhood lymphoblastic leukaemia: a randomised trial. Lancet. 2006 Oct 14;368(9544):1339–48.
5. Stork LC, Broxson EH, Sather H, Gaynon P, Matloub YH, Yanosfsky R, et al. Oral 6-thioguanine causes late-onset splenomegaly and portal hypertension in a subset of children with acute lymphoblastic leukemia. Gastroenterology. 2002;123(1):59.
6. Stoneham S, Lennard L, Coen P, Lilleyman J, Saha V. Veno-occlusive disease in patients receiving thiopurines during maintenance therapy for childhood acute lymphoblastic leukaemia. Br J Haematol. 2003 Oct;123(1):100–2.
7. Lancaster DL, Patel N, Lennard L, Lilleyman JS. 6-Thioguanine in children with acute lymphoblastic leukaemia: Influence of food on parent drug pharmacokinetics and 6-thioguanine nucleotide concentrations. Br J Clin Pharmacol. 2001;51(6):531–9.

8. Deibert P, Dilger K, Fischer C, Hofmann U, Nauck S, Stoelben S, et al. High variation of tioguanine absorption in patients with chronic active Crohn's disease. Aliment Pharmacol Ther. 2003;18(2):183–9.

9. van Asseldonk DP, de Boer NK, Smid K, Mulder CJ, van Bodegraven AA, Peters GJ. Limited intra-individual variability in hypoxanthine-guanine phosphoribosyl transferase, thiopurine S-methyl transferase, and xanthine oxidase activity in inflammatory bowel disease patients during 6-thioguanine therapy. Nucleosides, Nucleotides and Nucleic Acids. 2010;29(4–6):284–90.

10. Jharap B, de Boer NK, Vos RM, Smid K, Zwiers A, Peters GJ, et al. Biotransformation of 6-thioguanine in inflammatory bowel disease patients: A comparison of oral and intravenous administration of 6-thioguanine. Br J Pharmacol. 2011;163(4):722–31.

11. Jacobs SS, Stork LC, Bostrom BC, Hutchinson R, Holcenberg J, Reaman GH, et al. Substitution of oral and intravenous thioguanine for mercaptopurine in a treatment regimen for children with standard risk acute lymphoblastic leukemia: A collaborative Children's Oncology Group/National Cancer Institute pilot trial (CCG-1942). Pediatr Blood Cancer. 2007;49(3):250–5.

12. Wray L, Vujkovic M, McWilliams T, Cannon S, Devidas M, Stork L, et al. TPMT and MTHFR genotype is not associated with altered risk of thioguanine-related sinusoidal obstruction syndrome in pediatric acute lymphoblastic leukemia: a report from the Children's Oncology Group. Pediatr Blood Cancer. 2014 Nov;61(11):2086–8.

13. Roy Moulik N, M Taj M. Long-term risk of portal hypertension and related complications in children treated with 6-thioguanine for acute lymphoblastic leukemia: A single-center experience. Pediatr Blood Cancer. 2017 Oct;64(10):e26495.

14. Thomson B, Park JR, Felgenhauer J, Meshinchi S, Holcenberg J, Geyer JR, et al. Toxicity

and efficacy of intensive chemotherapy for children with acute lymphoblastic leukemia (ALL) after first bone marrow or extramedullary relapse. Pediatr Blood Cancer. 2004;43(5):571–9.

15. Herrlinger KR, Kreisel W, Schwab M, Fischer C, Scholmerich J, Fleig WE, et al. Open prospective trial an 6-thioguanine for chronic active Crohn's disease. Gastroenterology. 2002;122(4):A432.

16. Poordad, F.; Vasiliauskas, E.; Abreu, M.; Tran, T.; Martin, P.; Vierling, J.; Targan, S.; Geller, S.; Dubinsky MC. Nodular regenerative hyperplasia (NRH) of the liver associated with 6-thioguanine (6-TG) therapy in inflammatory bowel disease (IBD). Hepatology. 2002;36(4):333A.

17. Dubinsky MC, Vasiliauskas EA, Singh H, Abreu MT, Papadakis KA, Tran T, et al. 6-thioguanine can cause serious liver injury in inflammatory bowel disease patients. Gastroenterology. 2003 Aug;125(2):298–303.

18. Ferlitsch A, Teml A, Reinisch W, Ulbrich G, Wrba F, Homoncik M, et al. 6-Thioguanine associated nodular regenerative hyperplasia in patients with inflammatory bowel disease may induce portal hypertension. Am J Gastroenterol. 2007;102(11):2495–503.

19. Teml A, Schwab M, Hommes DW, Almer S, Lukas M, Feichtenschlager T, et al. A systematic survey evaluating 6-thioguanine-related hepatotoxicity in patients with inflammatory bowel disease. Wien Klin Wochenschr. 2007 Sep;119(17–18):519–26.

20. Pavlidis P, Ansari A, Duley J, Oancea I, Florin T. Splitting a therapeutic dose of thioguanine may avoid liver toxicity and be an efficacious treatment for severe inflammatory bowel disease: a 2-center observational cohort study. Inflamm Bowel Dis. 2014 Dec;20(12):2239–46.

21. van Asseldonk DP, Jharap B, Verheij J, den Hartog G, Westerveld DB, Becx MC, et al. The

Prevalence of Nodular Regenerative Hyperplasia in Inflammatory Bowel Disease Patients Treated with Thioguanine Is Not Associated with Clinically Significant Liver Disease. Inflamm Bowel Dis. 2016 Sep;22(9):2112–20.

22. Ward MG, Patel K V, Kariyawasam VC, Goel R, Warner B, Elliott TR, et al. Thioguanine in inflammatory bowel disease: Long-term efficacy and safety. United Eur Gastroenterol J. 2017 Jun;5(4):563–70.

23. De Bruyne R, Portmann B, Samyn M, Bansal S, Knisely A, Mieli-Vergani G, et al. Chronic liver disease related to 6-thioguanine in children with acute lymphoblastic leukaemia. J Hepatol. 2006 Feb;44(2):407–10.

24. Rawat D, Gillett PM, Devadason D, Wilson DC, McKiernan PJ. Long-term follow-up of children with 6-thioguanine-related chronic hepatoxicity following treatment for acute lymphoblastic leukaemia. J Pediatr Gastroenterol Nutr. 2011 Nov;53(5):478–9.

25. Broxson EH, Dole M, Wong R, Laya BF, Stork L. Portal hypertension develops in a subset of children with standard risk acute lymphoblastic leukemia treated with oral 6-thioguanine during maintenance therapy. Pediatr Blood Cancer. 2005 Mar;44(3):226–31.

26. Matloub Y, Lindemulder S, Gaynon P, Sather H, La M, Broxson E, et al. Intrathecal triple therapy decreases central nervous system relapse but fails to improve event-free survival when compared with intrathecal methotrexate: results of the Children’s Cancer Group (CCG) 1952 study for standard-risk acute lymphoblastic leukemi. Blood. 2006;1165–73.

27. Stork L, Erdmaru G, Adamson P. Oral 6-Thioguanine causes relatively mild and reversible hepatic veno-occlusive disease. J Pediatr Hematol Oncol. 1998;20:400.

28. Stork LC, Sather H, Hutchinson R, Broxson E, Matloub Y, Yanofsky R. Comparison of mercaptopurine (MP) with thioguanine (TG) and IT methotrexate (ITM) with IT “Triples” (ITT) in children with SR-ALL: results of CCG-1952. Vol. 100, Blood. 2002. p. 156a.

29. Erb N, Harms DO, Janka-Schaub G. Pharmacokinetics and metabolism of thiopurines in children with acute lymphoblastic leukemia receiving 6-thioguanine versus 6-mercaptopurine. Cancer Chemother Pharmacol. 1998 Aug 12;42(4):266–72.

30. Harms DO, Janka-Schaub GE, Grp CS. Co-operative study group for childhood acute lymphoblastic leukemia (COALL): long-term follow-up of trials 82, 85, 89 and 92. Leukemia. 2000;14(12):2234–9.

31. Lancaster DL, Lennard L, Rowland K, Vora AJ, Lilleyman JS. Thioguanine versus mercaptopurine for therapy of childhood lymphoblastic leukaemia: a comparison of haematological toxicity and drug metabolite concentrations. Br J Haematol. 1998 Jul;102(2):439–43.

32. Hann I, Vora A, Richards S, Hill F, Gibson B, Lilleyman J, et al. Benefit of intensified treatment for all children with acute lymphoblastic leukaemia: results from MRC UKALL XI and MRC ALL97 randomised trials. UK Medical Research Council's Working Party on Childhood Leukaemia. Leukemia. 2000;14(3):356–63.

33. Cartwright C, Vora A, Lennard L. Comparison of thiopurine dose intensity, thiopurine metabolism and neutropenia in children with acute lymphoblastic leukaemia taking thioguanine or mercaptopurine. Br J Clin Pharmacol. 2008;65(6):993–4.

34. Cartwright C, Vora A, Lennard L. Thiopurine methyltransferase activity in the clinical management of childhood acute lymphoblastic leukaemia. Br J Clin Pharmacol. 2011;987–8.

35. Lancaster D, Patel N, Lennard L, Lilleyman J. Leucocyte versus erythrocyte thioguanine nucleotide concentrations in children taking thiopurines for acute lymphoblastic leukaemia. Cancer Chemother Pharmacol. 2002;50(1):33–6.

36. Lennard L. Comparison of red and white cell 6-thioguanine nucleotide concentrations in children with lymphoblastic leukaemia on 6-mercaptopurine and 6-thioguanine therapy. Br J

Clin Pharmacol. 2001;52:476P.

37. Lennard L, Vora AJ, Kinsey S, Eden OB, Richards S, Lilleyman JS, et al. Thioguanine-related veno-occlusive disease (VOD) of the liver in children with acute lymphoblastic leukaemia (ALL). Br J Clin Pharmacol. 2003;55(4):437.

38. Lennard L, Richards SM, Mitchell CD, Cartwright C, Eden TO, Kinsey SE, et al. Thioguanine compared with mercaptopurine in childhood lymphoblastic leukaemia: Results of the United Kingdom medical research council randomised trial ALL97. Blood. 2005;106(11):255A.

39. Lennard L, Richards S, Cartwright CS, Mitchell C, Lilleyman JS, Vora A. The thiopurine methyltransferase genetic polymorphism is associated with thioguanine-related veno-occlusive disease of the liver in children with acute lymphoblastic leukemia. Clin Pharmacol Ther. 2006 Oct;80(4):375–83.

40. Lennard L, Cartwright C, Wade R, Richards S, Mitchell C, Kinsey S, et al. The clinical pharmacology of thioguanine vs. mercaptopurine in ALL97, a randomized trial for the treatment of childhood lymphoblastic leukaemia. Br J Clin Pharmacol. 2008;65(6):994.

41. Lennard L, Cartwright C, Wade R, Richards S, Mitchell C, Kinsey S, et al. Thioguanine versus mercaptopurine in ALL97: thiopurine metabolism and treatment outcome. Br J Haematol. 2008;141:32–3.

42. Lennard L, Cartwright CS, Wade R, Richards SM, Vora A. Thiopurine methyltransferase genotype-phenotype discordance and thiopurine active metabolite formation in childhood acute lymphoblastic leukaemia. Br J Clin Pharmacol. 2013;76(1):125–36.

43. Lennard L, Cartwright CS, Wade R, Vora A. Thiopurine dose intensity and treatment outcome in childhood lymphoblastic leukaemia: The influence of thiopurine methyltransferase pharmacogenetics. Br J Haematol. 2015;169(2):228–40.

44. Mitchell C, Kinsey S, Vora A, Richard S, Eden T. MRC ALL97/99 - A randomized comparison of dexamethasone versus prednisolone and thioguanine versus mercaptopurine in the treatment of childhood acute lymphoblastic leukemia. Blood. 2002;156a.

45. Mitchell C, Payne J, Wade R, Vora A, Kinsey S, Richards S, et al. The impact of risk stratification by early bone-marrow response in childhood lymphoblastic leukaemia: Results from the United Kingdom Medical Research Council trial ALL97 and ALL97/99. Br J Haematol. 2009;146(4):424–36.

46. Mitchell C, Richards S, Harrison CJ, Eden T. Long-term follow-up of the United Kingdom medical research council protocols for childhood acute lymphoblastic leukaemia, 1980-2001. Leukemia. 2010;24(2):406–18.

47. Vora A, Mitchell C, Kinsey S, Richards S, Eden T, Lilleyman J, et al. Thioguanine-related veno-occlusive disease (VOD) of the liver in children with acute lymphoblastic leukemia (ALL): report from United Kingdom Medical Research Council (UK MRC) trial ALL). Blood. 2002;100(11):36a.

48. Jacobs SS, Stork L, Bostrom BC, Hutchinson R, Holcenberg J, Reaman GH, et al. Treatment of newly diagnosed children with lower-risk leukemia: A collaborative Children's Cancer Group (CCG)/NCI pilot trial incorporating 6-thioguanine. J Clin Oncol. 2005;23(16):809S.

49. Lowe ES, Kitchen BJ, Erdmann G, Stork LC, Bostrom BC, Hutchinson R, et al. Plasma pharmacokinetics and cerebrospinal fluid penetration of thioguanine in children with acute lymphoblastic leukemia: a collaborative Pediatric Oncology Branch, NCI, and Children's Cancer Group study. Cancer Chemother Pharmacol. 2001 Mar 19;47(3):199–205.

50. Cheung TK, Florin THJ. 6-Thioguanine: A new old drug to procure remission in inflammatory bowel disease. Intern Med J. 2003;33:44–6.

51. Deibert P, Dilger K, Hofmann U, Nauck S, Stoelben S, Kreisel W. High variation of 6-

thioguanine absorption in patients with chronic active Crohn’s disease. Gastroenterology. 2003;124(4):A522–3.

52. Dubinsky MC, Hassard P V, Abreu MT, Kam LY, Seidman EG, Targan SR, et al. Thioguanine (6-TG): A therapeutic alternative in a subgroup of IBD patients failing 6-mercaptopurine (6-MP). Gastroenterology. 2000;118(4):A891.

53. Dubinsky MC, Feldman E, Abreu MT, Seidman EG, Baroni D, Kornbluth A, et al. Idiosyncratic adverse reactions to 6-mercaptopurine (6-MP) and azathioprine (AZA) can be averted by switching to thioguanine(G-TG) in patients with IBD. Gastroenterology. 2001;120(5):A12.

54. Dubinsky MC, Hassard P V, Seidman EG, Kam LY, Abreu MT, Targan SR, et al. An open-label pilot study using thioguanine as a therapeutic alternative in Crohn’s disease patients resistant to 6-mercaptopurine therapy. Inflamm Bowel Dis. 2001 Aug;7(3):181–9.

55. Dubinsky MC, Feldman EJ, Abreu MT, Targan SR, Vasiliauskas EA. Thioguanine: A potential alternate thiopurine for IBD patients allergic to 6-mercaptopurine or azathioprine. Am J Gastroenterol. 2003;98(5):1058–63.

56. Dubinsky MC, Vasiliauskas E, Singh H, Tran T, Papadakis K, Abreu M, et al. 6-thioguanine (6-TG) may cause serious liver injury in inflammatory bowel disease (IBD) patients. Gastroenterology. 2003;124(4):A8.

57. Geller SA, Poordad FF, Dubinsky MC, Cohen AH, Vasiliauskas EA, Abreu MT, et al. Hepatic nodular hyperplasia (sabourin lesion) and submicroscopic fibrosis associated with 6-thioguanine (6-TG) therapy in inflammatory bowel disease (IBD). Lab Investig. 2003;83(1):273A.

58. Geller SA, Dubinsky MC, Poordad FF, Vasiliauskas EA, Cohen AH, Abreu MT, et al. Early hepatic nodular hyperplasia and submicroscopic fibrosis associated with 6-thioguanine

therapy in inflammatory bowel disease. Am J Surg Pathol. 2004 Sep;28(9):1204–11.

59. Ferlitsch A, Teml A, Reinisch W, Wrba F, Ulbrich G, Ploner M, et al. 6-Thioguanine associated nodular regenerative hyperplasia in patients with inflammatory bowel disease may induce portal hypertension. Hepatology. 2005;42(4):511A–12A.

60. Hasyagar CP, Foster EN, Gerich M, Prindiville TP. Treatment of inflammatory bowel disease with 6-thioguanine (6-TG): Retrospective case series from a tertiary care center. Am J Gastroenterol. 2007;102:S490.

61. Ivastinovic D, Hoegenauer C, Petritsch W, Wenzl HH, Hinterleitner TA. Efficacy of 6-thioguanine in patients with Crohn’s disease intolerant to azathioprine. Gastroenterology. 2004;126(4):A630–1.

62. Jharap B, de Boer NK, Vos RM, Peters GJ, Wilhelm B, Mulder CJ, et al. Levels of 6-thioguanine nucleotides following oral and intravenous administration of relatively low-dosed 6-TG (0,3 mg/kg) in IBD-patients. Gastroenterology. 2008;134(4):A208.

63. Kornbluth A, Kissous M, George J, Dubinsky MC. “Lower” 6-thioguanine (6-TG) levels may be as effective as “higher” 6-TG levels in IBD patients treated with thioguanine. Gastroenterology. 2002;122(4):A221.

64. Omer OS, Salehi S, Pee L, Gera A, Loganayagam A. 6-Thioguanine as an alternative therapy in inflammatory bowel disease-experience in a London district general hospital. United Eur Gastroenterol J. 2015;1:A437.

65. Pavlidis P, Ansari AR, Duley J, Oancea I, Florin T. Splitting the normal daily dose of thioguanine may be efficacious treatment for IBD and avoid hepatic toxicity. Gastroenterology. 2014;1:S457.

66. Qasim A, McDonald S, Sebastian S, McLoughlin R, Buckley M, O’Connor H, et al. Efficacy and safety of 6-thioguanine in the management of inflammatory bowel disease. Scand J

Gastroenterol. 2007;42(2):194–9.

67. Saleem R, Gera A, Tranah TH, Paolino A, Loganayagam A. To evaluate the efficacy and safety of 6-thioguanine therapy in patients with inflammatory bowel disease-a London DGH experience. United Eur Gastroenterol J. 2013;1:A526.

68. Almer SH, Hjortswang H, Peterson C, Hindorf U. 6-thioguanine therapy in Crohn's disease - A Swedish experience. Gastroenterology. 2007;132(4):A185.

69. Almer SH, Hjortswang H, Hindorf U. 6-Thioguanine therapy in Crohn's disease--observational data in Swedish patients. Dig Liver Dis. 2009 Mar;41(3):194–200.

70. Bernstein C, Onken J. High erythrocyte 6-thioguanine (6-TG) levels are not associated with leukopenia in inflammatory bowel disease (IBD) patients treated with 6-TG. Gastroenterology. 2002;122(4):A221.

71. Bokemeyer B. Immunosuppressive therapy and biological therapeutics for the treatment of inflammatory bowel diseases (IBD). Verdauungskrankheiten. 2003;21(6):277–87.

72. Bonaz B, Boitard J, Marteau P, Lemann M, Belaiche J, Fournet J, et al. 6-thioguanine in patients intolerant to 6-mercaptopurine/azathioprine. Gastroenterology. 2002;122(4):A432.

73. Bonaz B, Boitard J, Marteau P, Lemann M, Coffin B, Flourie B, et al. Tioguanine in patients with Crohn's disease intolerant or resistant to azathioprine/mercaptopurine. Aliment Pharmacol Ther. 2003;18(4):401–8.

74. Herrlinger KR, Deibert P, Schwab M, Kreisel W, Fischer C, Fellermann K, et al. Remission maintenance by tioguanine in chronic active Crohn's disease. Aliment Pharmacol Ther. 2003;17(12):1459–64.

75. Herrlinger KR, Kreisel W, Deibert P, Schwab M, Fellermann K, Stange EF. 6-thioguanine maintains remission in chronic active Crohn's disease. Gastroenterology. 2003;124(4):A519.

76. Herrlinger KR, Kreisel W, Schwab M, Schoelmerich J, Fleig WE, Ruhl A, et al. 6-

Thioguanine - Efficacy and safety in chronic active Crohn’s disease. Aliment Pharmacol Ther. 2003;17(4):503–8.

77. Herrlinger KR, Fellermann K, Fischer C, Kreisel W, Deibert P, Schoelmerich J, et al. Thioguanine-nucleotides do not predict efficacy of tioguanine in Crohn’s disease. Aliment Pharmacol Ther. 2004;19(12):1269–76.

78. Klugmann T, Bokemeyer B. Long-term-tolerance of 6-thioguanine in outpatients with IBD. Verdauungskrankheiten. 2005;23(4):181–5.

79. Seiderer J, Reinisch W, Zech C, Diebold J, Wrba F, Harrer M, et al. Nodular regenerative hyperplasia in IBD patients on 6-thioguanine - a multicentre safety assessment by MRI and liver biopsy. Gastroenterology. 2004;126(4):A112.

80. Seiderer J, Zech CJ, Reinisch W, Lukas M, Diebold J, Wrba F, et al. A multicenter assessment of liver toxicity by MRI and biopsy in IBD patients on 6-thioguanine. J Hepatol. 2005 Aug;43(2):303–9.

81. Teml A, Harrer M, Schwab M, Dejaco C, Mantl M, Vogelsang H, et al. Safety and efficacy of 6-thioguanine (6-TG) in patients with ulcerative and indeterminate colitis intolerant or resistant to azathioprine and/or 6-mercaptopurine (Aza/6-MP). Gastroenterology. 2003;124(4):A524.

82. Teml A, Schwab M, Harrer M, Miehsler W, Schaeffeler E, Dejaco C, et al. A prospective, open-label trial of 6-thioguanine in patients with ulcerative or indeterminate colitis. Scand J Gastroenterol. 2005;40(10):1205–13.

83. Teml A, Dejaco C, Miehsler W, Harrer M, Vogelsang H, Reinisch W. Long-term efficacy of 6-thioguanine in patients with inflammatory bowel disease. Gastroenterology. 2005;128(4):A587.

84. Zech CJ, Seiderer J, Reinisch W, Ochsenkuhn T, Schima W, Diebold J, et al. Thioguanin-

induced nodular regenerative hyperplasia of the liver-ROC analysis of different MR techniques. Eur Radiol. 2007;17(7):1898–905.

85. Harrer M, Schwab M, Teml A, Dejaco C, Mantl M, Vogelsang H, et al. Open prospective study on 6-thioguanine (6-TG) for Crohn's disease (CD) intolerant or resistant to azathioprine (AZA) and/or 6-mercaptopurine (6-MP). Gastroenterology. 2003;124(4):A521.

86. Piel B, Vaidya S, Lancaster D, Taj M, Pritchard-Jones K, Stoneham S, et al. Chronic hepatotoxicity following 6-thioguanine therapy for childhood acute lymphoblastic leukaemia [4] (multiple letters). Br J Haematol. 2004;125(3):410–2.

87. Ramanujachar R, Richards S, Hann I, Goldstone A, Mitchell C, Vora A, et al. Adolescents with acute lymphoblastic leukaemia: outcome on UK national paediatric (ALL97) and adult (UKALLXII/E2993) trials. Pediatr Blood Cancer. 2007;48(3):254–61.

88. Ravikumara M. 6-thioguanine related hepatotoxicity in children with acute lymphoblastic leukaemia - A dual centre experience. J Pediatr Gastroenterol Nutr. 2005;40(5):677.

89. Ravikumara M, Hill FGH, Wilson DC, Gillett PM, Thomas A, Brown R, et al. 6-Thioguanine-related chronic hepatotoxicity and variceal haemorrhage in children treated for acute lymphoblastic leukaemia - A dual-centre experience. J Pediatr Gastroenterol Nutr. 2006;42(5):535–8.

90. Roy Moulik N, Potter E, Height S, Vaidya S, Lancaster D, Taj M. Long term follow-up of 6-thioguanine-induced hepatotoxicity in children treated on UKALL 97/99 protocol; the royal Marsden experience. Pediatr Blood Cancer. 2015;62:S259.

91. de Boer NK, de Graaf P, Wilhelm AJ, Mulder CJJ, van Bodegraven AA. On the limitation of 6-tioguaninenucleotide monitoring during tioguanine treatment. Aliment Pharmacol Ther. 2005;22(5):447–51.

92. de Boer NK, Derijks LJ, Gilissen LP, Hommes DW, Engels LG, De-Boer SY, et al. On

tolerability and safety of a maintenance treatment with 6-thioguanine in azathioprine or 6-mercaptopurine intolerant IBD patients. World J Gastroenterol. 2005 Sep 21;11(35):5540–4.

93. de Boer NK, Derijks LJ, Gilissen LP, Hommes DW, Engels LG, de Boer S, et al. Maintenance treatment with 6-thioguanine in azathioprine or 6-mercaptopurine intolerant IBD patients. Gastroenterology. 2005;128(4):A199.

94. de Boer NK, Derijks LJ, Keizer-Garritsen JJ, Lambooy LH, Ruitenbeek W, Hooymans PM, et al. Extended thiopurine metabolite assessment during 6-thioguanine therapy for immunomodulation in crohn's disease. J Clin Pharmacol. 2007;47(2):187–91.

95. de Boer NK, Gilissen LP, Derijks LJ, den Hartog G, Westerveld BD, Engels L, et al. Hepatotoxicity of long-term and low-dose 6-thioguanine in IBD patients. Eur J Gastroenterol Hepatol. 2007;19(10):A20–1.

96. de Boer NK, Zondervan PE, Gilissen LP, den Hartog G, Westerveld BD, Derijks LJJ, et al. Absence of nodular regenerative hyperplasia after low-dose 6-thioguanine maintenance therapy in inflammatory bowel disease patients. Dig liver Dis. 2008 Feb;40(2):108–13.

97. De Jong D, Derijks L, Engels L, Jansen J, Hooymans P, Mulder C. 6-thioguanine seems promising in azathioprine intolerant patients. Gastroenterology. 2002;122(4):A432.

98. Derijks LJ, De Jong DJ, Gilissen LP, Engels L, Mulder CJ, Bos LP, et al. 6-Thioguanine in azathioprine and 6-mercaptopurine intolerant patients with inflammatory bowel disease. Br J Clin Pharmacol. 2002;53(5):546P–7P.

99. Derijks LJ, De Jong DJ, Gilissen LP, Engels LG, Hooymans PM, Jansen JB, et al. 6-Thioguanine seems promising in azathioprine- or 6-mercaptopurine-intolerant inflammatory bowel disease patients: A short-term safety assessment. Eur J Gastroenterol Hepatol. 2003;15(1):63–7.

100. Derijks LJ, Gilissen LP, Engels LG, Bos LP, Bus PJ, Lohman JJ, et al. Pharmacokinetics of

6-thioguanine in patients with inflammatory bowel disease. Ther Drug Monit. 2006 Feb;28(1):45–50.

101. Gilissen LPL, Derijks LJJ, Driessen A, Bos LP, Hooymans PM, Stockbrügger RW, et al. Toxicity of 6-thioguanine: no hepatotoxicity in a series of IBD patients treated with long-term, low dose 6-thioguanine. Some evidence for dose or metabolite level dependent effects? Dig Liver Dis. 2007 Feb;39(2):156–9.

102. Jharap B, Mulder CJ, den Hartog G, Westerveld BD, Engels LG, de Jong DJ, et al. Adequately dosed 6-TG use in chronic intestinal inflammation is not associated with nodular regenerative hyperplasia: A series of 73 liver biopsies. Gastroenterology. 2007;132(4):A179.

103. van Asseldonk DP, Jharap B, de Boer NK, Mulder CJ, van Bodegraven AA. 6-Thioguanine induces remission and mucosal healing in IBD. Gastroenterology. 2009;1:A656.

104. van Asseldonk DP, Jharap B, de Boer NK, van Bodegraven AA, Mulder CJ. Adequately dosed 6-thioguanine is a well tolerated and safe rescue drug in azathioprine or 6-mercaptopurine intolerant IBD patients. Gastroenterology. 2009;1:A654.

105. van Asseldonk DP, Jharap B, de Boer NK, Zondervan PE, Bloemena E, Den Hartog G, et al. Liver histology of IBD patients who are treated with 6-thioguanine due to failure of conventional thiopurines reveals very few cases of nodular regenerative hyperplasia. Gastroenterology. 2010;1:S62.

106. van Asseldonk DP, van Bodegraven AA, Mulder CJ. Azathioprine or 6-mercaptopurine associated hepatotoxicity diminishes upon administration of 6-thioguanine in IBD patients. Gastroenterology. 2010;1:S674.

107. van Asseldonk DP, Jharap B, Kuik DJ, de Boer NK, Westerveld BD, Russel MG, et al. Prolonged thioguanine therapy is well tolerated and safe in the treatment of ulcerative colitis. Dig Liver Dis. 2011 Feb;43(2):110–5.

108. van Asseldonk DP, Seinen ML, de Boer NK, van Bodegraven AA, Mulder CJ. Hepatotoxicity associated with 6-methyl mercaptopurine formation during azathioprine and 6-mercaptopurine therapy does not occur on the short-term during 6-thioguanine therapy in IBD treatment. J Crohns Colitis. 2012;6(1):95–101.

109. van Asseldonk D, Jharap B, de Boer NK, Verheij J, den Hartog G, Russel MG, et al. No Nodular Regenerative Hyperplasia in Sequential Liver Biopsies During Tioguanine Maintenance Therapy in Inflammatory Bowel Disease Patients. Gastroenterology. 2014;146(5):S580–1.

110. Ansari A, Elliott T, Fong F, Arenas-Hernandez M, Rottenberg G, Portmann B, et al. Further experience with the use of 6-thioguanine in patients with Crohn's disease. Inflamm Bowel Dis. 2008 Oct;14(10):1399–405.

111. Elliott T, Ansari A, Soon S, Fong F, Baburajan B, Arenas-Hernandez M, et al. Further experience with the use of 6-thioguanine as an alternative immunosuppressant in patients with crohn's disease. Gut. 2007;56:A23.

112. Patel K, Goel R, Kariyawasam V, Blaker P, Ward M, Ray S, et al. Thioguanine remains an acceptable alternative immunosuppressant in IBD. United Eur Gastroenterol J. 2013;1:A64.

113. Soon SY, Ansari A, Fong FM, Hirst J, Sanderson JD. 6-thioguanine is well tolerated and reasonably effective in Crohn's disease patients who are resistant or intolerant of azathioprine and methotrexate. Gut. 2004;53:A104.

114. Ward MG, Kariyawasam VC, Patel K V, Elliot TR, Blaker PA, Mogan SB, et al. Thioguanine use in inflammatory bowel disease: 13 year experience in a tertiary centre. J Gastroenterol Hepatol. 2013;2:99–100.

115. Bisschop D, Germain ML, Munzer M, Trenque T. [Thioguanine, pancreatotoxicity?]. Therapie. 2001;56(1):67–9.

116. Broxson EH, Dole M, Wong R, Laya BF, Stork LC. Portal hypertension (PH) develops in a subset of children treated with maintenance oral 6 thioguanine (TG) for standard risk acute lymphoblastic leukemia (SR-ALL). Blood. 2001;98(11):113A.

117. Chojnacki C, Romanowski M, Wachowska-Kelly P. Psychosomatic complications during treatment for ulcerative colitis. Gastroenterol Rev. 2012;1(1):52–5.

118. de Boer NK, Mulder CJJ, van Bodegraven AA. Nodular regenerative hyperplasia and thiopurines: the case for level-dependent toxicity. Liver Transplant. 2005 Oct;11(10):1300–1.

119. Fritz E, Wrba F, Knoflach P. Variceal hemorrhage in a patient with ulcerative colitis treated with 6-thioguanine. Inflamm Bowel Dis. 2008;14(4):582–3.

120. Kane S, Cohen SM, Hart J. Acute sinusoidal obstruction syndrome after 6-thioguanine therapy for Crohn’s disease. Inflamm Bowel Dis. 2004 Sep;10(5):652–4.

121. Mao J, Zhao F, Song H, Yang S, Shen D, Tang Y. Thioguanine treatment-related sinusoidal obstruction syndrome in 2 children. Zhongguo Dang Dai Er Ke Za Zhi. 2013;15(9):788–90.

122. Marasco G, Scaioli E, Renzulli M, Colecchia A, Golfieri R, Festi D, et al. MRI Patterns in a Case of 6-Thioguanine-Related Hepatic Sinusoidal Obstruction Syndrome. Am J Gastroenterol. 2016;111(6):767.

123. Mares WG, Wong DR, Gilissen LP, Masclee AA, Hooymans PM, Engels LG. Safe 6-thioguanine therapy of a TPMT deficient Crohn’s disease patient by using therapeutic drug monitoring. J Crohn’s Colitis. 2009;3(2):128–30.

124. Wong DR, Mares WGN, Gilissen LPL, Masclee AAM, Engels L, Neef C, et al. Safe and effective 6-thioguanine treatment of a thiopurine S-methyltransferase deficient Crohn’s disease patient using therapeutic drug monitoring. Br J Clin Pharmacol. 2009;68(1):138.

125. Merino JM, Casanova F, Saez-Royuela F, Velasco A, Gonzalez JB. Veno-occlusive disease of the liver associated with thiopurines in a child with acute lymphoblastic leukemia. Pediatr

Hematol Oncol. 2000;17(5):429–31.

126. Nielsen SN, Frandsen TL, Nersting J, Hjalgrim LL, Schmiegelow K. Pharmacokinetics of 6-thioguanine and 6-mercaptopurine combination maintenance therapy of childhood ALL: Hypothesis and case report. J Pediatr Hematol Oncol. 2015;37(3):e206–9.

127. Radys, W.; Kaminska, B.; Landowski, P.; Brodzicki J. The case of colitis ulcerosa - Diagnostic and therapeutic difficulties. Pediatr Wspolczesna. 2006;8(2):131–3.

128. Rulyak SJ, Saunders MD, Lee SD. Hepatotoxicity associated with 6-thioguanine therapy for Crohn's disease. J Clin Gastroenterol. 2003;36(3 PG-234-237):234–7.

129. Salerno A, Vacante M, Pollina D, Stancanelli B, Martini S, David E, et al. Liver veno-occlusive disease (VOD) in a patient given 6-thioguanine for crohn's disease. Br J Med Pract. 2014;7(2):a711.

130. Shastri S, Dubinsky MC, Poordad F, Vasiliauskas E, Geller SA. Nodular hyperplasia of the liver (Sabourin lesion) occurring with inflammatory bowel diseases in association with 6-thioguanine therapy. Am J Clin Pathol. 2002;118(4):631.

131. Shastri S, Dubinsky MC, Fred Poordad F, Vasiliauskas EA, Geller SA. Early nodular hyperplasia of the liver occurring with inflammatory bowel diseases in association with thioguanine therapy. Arch Pathol Lab Med. 2004;128(1):49–53.

132. de Boer NK, van Elburg RM, Wilhelm AJ, Remmink AJ, van Vugt JM, Mulder CJ, et al. 6-Thioguanine for Crohn's disease during pregnancy: Thiopurine metabolite measurements in both mother and child. Scand J Gastroenterol. 2005;40(11):1374–7.

133. Van den Berg SA, de Boer M, van der Meulen-de Jong AE, Jansen JM, Hoentjen F, Russel MG, et al. Safety of Tioguanine During Pregnancy in Inflammatory Bowel Disease. J Crohns Colitis. 2016;10(2):159–65.

134. Wenzl HH, Hogenauer C, Fickert P, Petritsch W. Thioguanine-induced symptomatic

thrombocytopenia. Am J Gastroenterol. 2004;99(6):1195.
