# Supplementary material for: Hepatotoxicity during 6-thioguanine treatment in inflammatory bowel disease and childhood acute lymphoblastic leukaemia: A systematic review
Source: PLoS One. 2019 May 24;14(5):e0212157. doi: 10.1371/journal.pone.0212157 (PMC6534292; doi:10.1371/journal.pone.0212157)
Supplement: S4 Appendix — (PDF) [file pone.0212157.s004.pdf]

S4 Appendix. Risk of bias of included randomised controlled trials

	Random sequence generation (selection bias)	Allocation concealment (selection bias)	Blinding of participants and personnel (performance bias)	Blinding of outcome assessment (detection bias)	Incomplete outcome data (attrition bias)	Selective reporting (reporting bias)	Other bias
ALL-REZ BFM 87	?	?	-	+	+	+	?
CCG-1952	+	+	-	-	+	+	?
COALL-92	?	-	-	-	+	+	?
UK MRC ALL97/99	+	+	-	-	+	+	?

Judgements of each risk of bias item for each included randomised controlled trial. The items assessed included: (1) sequence generation, (2) allocation concealment, (3) blinding of participants and personnel, (4) blinding of outcome assessors, (5) incomplete outcome data, (6) selective outcome reporting, and (7) other bias including baseline imbalance, early stopping, bias due to financial interest, and academic bias.

- high risk; ? unclear risk; + low risk.
